# Long-Term Sorption of Metals Is Similar among Plastic Types: Implications for Plastic Debris in Aquatic Environments

**DOI:** 10.1371/journal.pone.0085433

**Published:** 2014-01-15

**Authors:** Chelsea M. Rochman, Brian T. Hentschel, Swee J. Teh

**Affiliations:** 1 Department of Biology and Coastal Marine Institute, San Diego State University, San Diego, California, United States of America; 2 Department of Anatomy, Physiology and Cell Biology, School of Veterinary Medicine at the University of California Davis, Davis, California, United States of America; Northwest Fisheries Science Center, NOAA Fisheries, United States of America

## Abstract

Concerns regarding plastic debris and its ability to accumulate large concentrations of priority pollutants in the aquatic environment led us to quantify relationships between different types of mass-produced plastic and metals in seawater. At three locations in San Diego Bay, we measured the accumulation of nine targeted metals (aluminum, chromium, manganese, iron, cobalt, nickel, zinc, cadmium and lead) sampling at 1, 3, 6, 9 and 12 months, to five plastic types: polyethylene terephthalate (PET), high-density polyethylene (HDPE), polyvinyl chloride (PVC), low-density polyethylene (LDPE), and polypropylene (PP). Accumulation patterns were not consistent over space and time, and in general all types of plastic tended to accumulate similar concentrations of metals. When we did observe significant differences among concentrations of metals at a single sampling period or location in San Diego Bay, we found that HDPE typically accumulated lesser concentrations of metals than the other four polymers. Furthermore, over the 12-month study period, concentrations of all metals increased over time, and chromium, manganese, cobalt, nickel, zinc and lead did not reach saturation on at least one plastic type during the entire 12-month exposure. This suggests that plastic debris may accumulate greater concentrations of metals the longer it remains at sea. Overall, our work shows that a complex mixture of metals, including those listed as priority pollutants by the US EPA (Cd, Ni, Zn and Pb), can be found on plastic debris composed of various plastic types.

## Introduction

Plastic debris litters the aquatic environment globally [1], [2], contaminating a diversity of habitats and organisms. For example, coral reefs [3] and salt marshes [4] can be smothered and/or entangled by plastic debris, and animals from all seven species of seaturtles and 45% of all species of marine mammals have been found with plastic in their digestive tracts [5]. The physical hazards of plastic debris to organisms include ingestion, entanglement and smothering [6], [7]. The chemical effects associated with the ingestion or bioconcentration of plastic debris have received less attention, despite the ingestion of plastic by hundreds of species [5].

Plastic debris is associated with a “cocktail of contaminants” made up of chemical ingredients in the plastic and chemical pollutants sorbed to the plastic from the environment [8], including 78% of chemicals listed as ‘priority pollutants’ (chemicals prioritized by the US EPA because they have been found to be bioaccumulative, persistent and/or toxic; [9]). Greater than half of polymers produced include chemical ingredients considered hazardous by the UN's Globally Harmonized System [10]. Moreover, when discarded into aquatic environments, plastic debris sorbs chemical contaminants from surrounding water (some up to 100 × that of sediments [11]), including organic chemicals that are persistent, bioaccumulative and toxic (PBTs [12], [13]) and metals [14], [15]. Thus, plastic debris potentially acts as a multiple stressor to organisms [8], inflicting both physical and chemical adverse effects to animals upon ingestion. As such, efforts to manage plastic debris should understand the complex mixture of contaminants associated with this material when its fate is the aquatic environment.

The presence of organic chemical pollutants on plastic debris is established globally [12], [16], [17], but the presence of metals on plastic debris has only recently been studied [14], [15], [18]. Like many chemical pollutants (e.g. polybrominated diphenyl ethers [19]), some metals found on plastic debris (e.g., lead; [18]) derive from the manufacturing process and from environmental sorption [14], [15]. But, like several organic chemical pollutants (e.g. DDT [12]), several metals are found on plastic debris solely as a result of environmental sorption [14], [15]. Environmental accumulation may have been expected, as the surfaces of plastic containers are known to accumulate metals from water samples [20], [21], [22]. To investigate this further, a recent study deployed virgin pellets of polyethylene in an urban harbor for 8 weeks and found several metals, as well as organic and inorganic precipitates on the weathered surfaces of pellets, but the time course of metal accumulation was not measured [14]. Unexpectedly, analysis of individual polyethylene resin pellets collected from beaches in southwest England contained similar concentrations of metals as local sediment—which is striking because sediment generally has a much greater surface area [15]. Chemical ingredients in plastics from manufacturing (e.g., catalysts, fillers, plasticizers) [22] and degradation and fouling of aquatic plastic debris via microbial biofilms and colonization by algae and invertebrates [15], [23] may generate active sites for the sorption and/or bioaccumulation of metals.

Plastic debris is composed of several different polymers, and their unique chemical ingredients may make some types of plastic more hazardous than others when their chemical constituents are bioavailable to organisms. For example, polyvinyl chloride (PVC), polycarbonate, polyurethane and polystyrene (PS) are composed of hazardous monomers (e.g., vinyl chloride, bisphenol-A and styrene) and/or contain hazardous additives (e.g. PBDEs, phthalates and lead) [9]. For other plastics, their chemical properties may facilitate sorption of large concentrations of contaminants, enhancing their toxicity. For example, polyethylene and polypropylene (PP) sorb an order of magnitude greater concentration of polychlorinated biphenyls (PCBs) than polyethylene terephthalate (PET) and PVC [13]. Moreover, concentrations of hazardous chemicals may vary based on the location where the material is discarded and the time it is left in the aquatic environment. Plastics recovered from locations with greater chemical contamination have greater concentrations of chemicals sorbed to them [12], [13]. Over time, plastics degrade and foul with life [24], [25], increasing the surface area and changing the surface properties, allowing concentrations of chemical contaminants to increase over time [13], [15], [26] via sorption and/or bioaccumulation by biofilms. Thus, assessing the hazards associated with plastic in aquatic habitats requires knowledge of the types of polymers comprising the debris, the length of time the debris was present in the aquatic environment (affecting the size, shape and fouling) and the locations and transport of the debris during that time period.

Plastic pre-production pellets, a recognizable component of marine debris, have been used to establish a global association between organic contaminants and plastic discarded at sea [12]. Here, we assess the accumulation of inorganic contaminants (i.e. metals) to plastic pellets. The physical and chemical properties of each type of plastic (e.g. surface area [11], diffusivity [26] [27] [28] [29] and crystallinity [26] [29]) influence the sorption of chemicals to plastic debris [27] [28]. Thus, we deployed different types of plastic pre-production pellets in an urban bay to measure how the accumulation of various metals varies among different types of plastic in the marine environment.

**Figure 1:**
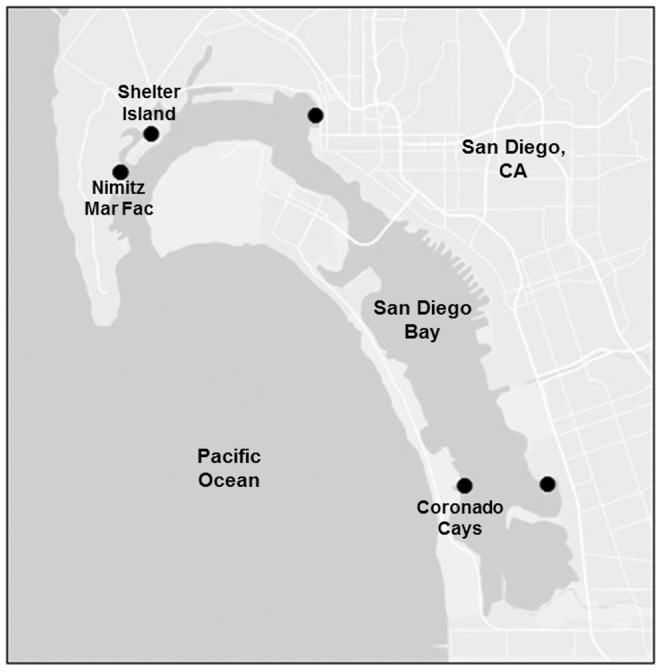
Map of San Diego Bay. The map shows the three study locations: Coronado Cays, Shelter Island and Nimitz Marine Facility. Figure generated with ArcGIS version 9.3.

We aimed to measure the concentrations of pollutants over a 12-month period, and achieved this by deploying five of the most common types of mass-produced pre-production plastic pellets [30] at multiple locations in an urban bay. Laboratory experiments accurately apply a dose of contaminants to polymers and hold many confounding variables constant (e.g. controlling for temperature and fouling). In contrast, our field experiment incorporated environmentally relevant factors and allowed the polymers to be exposed to *in situ* variations in all variables relevant at our field sites, including fluctuating *in situ* concentrations of metals and the growth of biofilms. Our previous work showed that concentrations of organic pollutants (polycyclic aromatic hydrocarbons and PCBs) sorbed to plastics varied significantly among polymers, locations and length of time in the bay [13]. Here, we report the first long-term measurement of the accumulation of metals to these five types of plastics. We measured aluminum (Al), chromium (Cr), manganese (Mn), iron (Fe), cobalt (Co), nickel (Ni), zinc (Zn), cadmium (Cd) and lead (Pb) to PET, high-density polyethylene (HDPE), PVC, low-density polyethylene (LDPE) and PP. We deployed two independent replicates of uncontaminated, virgin pre-production pellets of each polymer at three locations in San Diego Bay, CA, USA (Figure 1) and recovered them after 1, 3, 6, 9 or 12 months. We hypothesized that the accumulation of metals would vary among plastic type and locations in San Diego Bay. Although previous laboratory experiments, measuring sorption kinetics using polyethylene, found that metals reached equilibrium within 25-100 hours [15], [20], we expected to measure increasing concentrations during at least the first several months of the experiment. In the field, plastic weathers [13], [14], [15] and the sorption of metals is predicted to be enhanced as the polarity, surface area and porosity of the plastic increase and become fouled with organic matter and hydrous metal oxides [14].

## Methods

### Ethics Statement

On June 1^st^, 2009 five types of pre-production plastic pellets, PET, HDPE, PVC, LDPE and PP (denoted by recycle codes 1–5, respectively, in the USA), were deployed from docks at three locations throughout San Diego Bay (Figure 1): Coronado Cays, Shelter Island and Nimitz Marine Facility. Each of these docks is located at a privately owned facility and permission was granted to conduct the study at each location. Coronado Cays and Shelter Island are floating docks and Nimitz Marine Facility is a fixed-elevation dock.

### Experimental Design

At each location, two replicate samples (5 g) of each plastic type were deployed for future collection at the end of five time periods: 1, 3, 6, 9, and 12 months (150 total samples). Each replicate consisted of 5 g of pellets of one plastic type placed in individual Nitex mesh (1.3 mm) bags. Pellets of PET were cylindrical (3 mm long, 2 mm diameter), and pellets of HDPE, PVC, LDPE, and PP were spherical (3 mm diameter). To facilitate removal of large fouling organisms (e.g., tunicates and bivalves), each Nitex bag was placed inside a nylon mesh (10 mm) bag. Replicate samples were deployed by hanging each nylon bag on one of two identical PVC frames suspended from each dock (see Figure S1 for a schematic diagram). Each frame contained 25 sets of bags containing samples of plastic, one randomly assigned replicate of each of the 25 combinations of 5 plastic types and 5 time periods. On each dock, the two PVC frames were positioned approximately 2 meters apart, and we assumed that variation between replicates is not related to differences between the two frames used to hang the bags. Bags containing plastic samples were suspended at a depth approximately 0.5 m below the surface of floating docks or 0.5 m below Mean Lower Low Water at Nimitz Marine Facility (a fixed-elevation dock). Every 2 weeks, fouling organisms were scrubbed from the outer nylon bags to maintain flow of water to the pellets. At the end of their randomly assigned deployment time, the two replicate samples of each type of plastic were collected and the outer nylon bag was removed. Immediately, each individual nitex bag of pellets was wrapped in clean foil and placed in clean polyethylene bags. Samples were stored at −20°C until analysis.

### Materials and Reagents

Unless stated, reagents were purchased from Fisher Scientific (Fair Lawn, NJ, USA) and were of analytical grade or better. All plasticware used for sample processing was rinsed five times with each 10% hydrochloric acid (HCl) followed by Millipore Milli-Q water. After drying under a laminar flow hood, clean plasticware was stored in polyethylene bags until use.

### Sample Digestion

Pellets from each field-collected Nitex bag were rinsed in ultrapure water to remove sediment. In a few cases, large fouling organisms were seen attached to individual pellets; these pellets were excluded from chemical analyses. The frequency at which individual pellets were observed to be fouled did not vary noticeably among samples from different deployment times or from different sampling locations. Thirty pellets from each sample were weighed into individual polypropylene centrifuge tubes. Two milliliters (mL) of 20% aqua regia (1 part nitric acid (HNO_3_) to 3 parts HCl) were added to each sample and centrifuge tubes were screw-capped. Samples were then agitated for 24 hours at room temperature using a Roto-Shake Genie (Scientific Industries, USA) before 1 mL of digested solution was transferred into a clean tube and diluted with 9 milliliters Millipore Milli-Q water for analysis. Virgin pellets of each type were used as laboratory blanks and 0-month controls (n = 3).

### Metal Analysis

The prepared samples were analyzed for Al, Cr, Mn, Fe, Co, Ni, Zn, Cd and Pb by the Interdisciplinary Center for Plasma Mass Spectrometry at the University of California at Davis (ICPMS.UCDavis.edu) using an Agilent 7500CE ICP-MS (Agilent Technologies, Palo Alto, CA). Although concentrations of copper are of widespread interest in urban marine systems, concentrations of copper were inadvertently not measured by the analytical laboratory used in our study. The samples were introduced using a MicroMist Nebulizer (Glass Expansion, 4 Barlow's Landing Rd., Unit 2A Pocasset, MA 02559) into a temperature controlled spray chamber with helium as the Collision Cell gas. Instrument standards were diluted from Certiprep ME 2A (SPEX CertiPrep, 203 Norcross Avenue, Metuchen, NJ 08840) to 0.5, 1, 10, 100, 200, 500, 1000, 2000 and 5000 µg/L respectively in 3% Trace Element HNO_3_ (Fisher Scientific) in 18.2 Megaohm-cm water. A NIST 1643E Standard (National Institute of Standards and Technology, 100 Bureau Drive, Stop 2300, Gaithersburg, MD 20899-2300) was analyzed initially and QC standards consisting of a ME 2A at a concentration of 100 µg/L were analyzed every 12^th^ sample as quality controls. Sc, Y, and Bi Certiprep standards (SPEX CertiPrep) were diluted to 100 µg/L in 3% HNO_3_ and introduced by peristaltic pump as an internal standard.

### Statistical Methods

Average levels of metals measured in laboratory blanks (i.e. time-zero controls that were never deployed in the field) were subtracted from the reported concentrations of metals extracted from pellet samples. Statistical analyses were performed using GMAV (GMAV; EICC, University of Sydney). We decided *a priori* to test for differences among plastic types and locations by performing a 2-factor ANOVA (α = 0.05) on data from each sampling period individually. When the interaction term was p>0.250, this term was pooled [31], [32], [33]. Concentrations of all metals were (x+0.1) log transformed to achieve normality [32]. Homogeneity of variance was verified by Cochran's (1951) *C-*test. *Post-hoc* Student-Newman-Keuls (SNK) tests were used to distinguish significantly different treatment means (α = 0.05). We decided *a priori* to quantify temporal patterns by fitting a pseudo-first-order kinetics model [34] to the concentrations accumulated on each type of plastic at each location in the bay. We expected accumulation patterns to follow a pseudo-first-order kinetics model as it does for several metals on numerous materials including silica, algae and crab shell particles [34] and because the accumulation of metals to plastic pellets consists of a period of rapid adsorption followed by a slower approach to equilibrium [15]. SigmaPlot 10 (SYSTAT Software, Chicago, IL) was used to fit the exponential rise to maximum equation C_t_ = C_eq_(1-e^−kt^), where C_t_ is the concentration at time t, C_eq_ is the predicted equilibrium concentration, and k is the rate constant.

## Results

**Figure 2:**
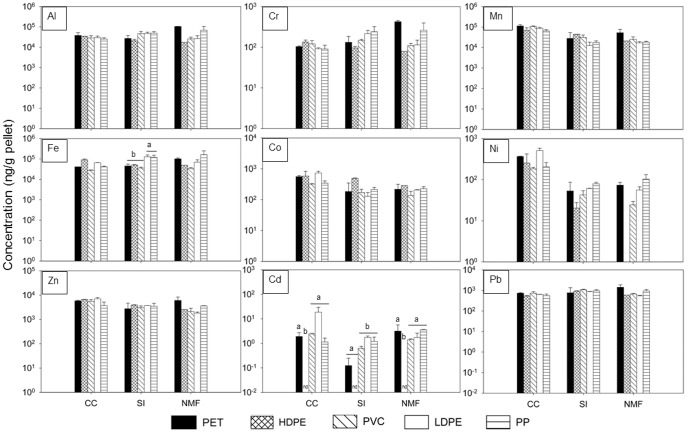
Metal concentrations (ng/g) for each plastic type after 12 months in San Diego Bay. Concentrations of metals are shown for each of the five plastic types (PET-black, HDPE-hatched, PVC-diagonal stripes, LDPE-white, PP-horizontal stripes) at each of the three sites (CC-Cornado Cays, SI-Shelter Island, NMF-Nimitz Marine Facility) ordered from the back to the front of the bay. Each graph represents one of the 9 targeted metals (Al, Cr, Mn, Fe, Co, Ni, Zn, Cd, Pb) ordered from left to right according to molecular weight. Each bar represents the mean concentration (ng/g) + standard error (n = 2). A non-detect is denoted by nd. ANOVA showed statistically significant differences among plastic types (*P*<0.05)* for Fe and Cd.

All nine targeted metals were detected and quantified on all polymer types, with the exception of Cd on HDPE (Figures 2 and S9; see Table S1 for concentrations of all targeted metals on each individual sample). In contrast to what we observed for organic contaminants [13], the concentrations of metals that accumulated on plastic pellets did not vary consistently among polymers, except for Cr, Mn and Co where concentrations were similar among all polymers at all locations and sampling periods (Figures 2, S3, S4 and S6). For the other six metals analyzed (Figures S2, S5, S7, S8 and S10), 2-factor ANOVAs showed that, in general, differences among polymers often varied according to location and sampling period (see Table S2 for ANOVA results showing accumulation patterns for each metal at each time period and location). For Al and Fe, although there was a significant polymer x location interaction at two out of the five time periods, SNK comparisons did not reveal a general trend among polymers (Table S2). Still, for Ni, Zn, Cd and Pb, where there were significant differences among polymers and/or a significant polymer x location interaction, SNK comparisons revealed that metal concentrations were smaller on HDPE than on other polymer types with the exception of Ni sampled at 12 months from Coronado Cays where PVC accumulated the smallest concentrations of Ni (Table S2).

For all metals, concentrations accumulated on plastics varied significantly among locations at a minimum of three out of the five time periods. For Al, Cr, Fe and Pb, concentrations were greatest at Shelter Island and Nimitz Marine Facility closer to the mouth of the bay (Table S2). For Mn, Co, Ni, Zn and Cd concentrations were greatest at Coronado Cays in the back of the bay (Table S2).

**Figure 3:**
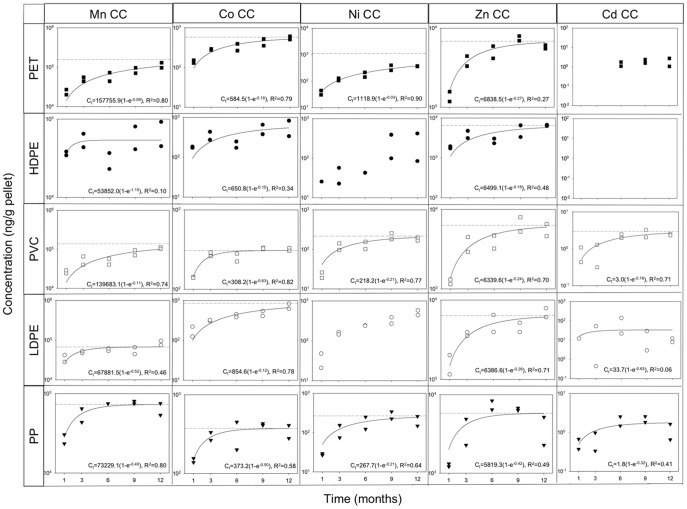
Concentrations of Mn, Co, Ni, Zn and Cd over time. Concentrations of Mn, Co, Ni, Zn and Cd (ng/g of pellets) vs time for each type of plastic at Coronado Cays (CC) where contamination was greatest. Rows represent plastic types PET, HDPE, PVC, LDPE and PP (in order from top to bottom). Columns represent metals ordered from left to right according to molecular weight. Note that vertical axes differ among graphs. Data were fit to the first-order approach to equilibrium model [26] using the exponential rise to maximum equation C_t_  =  C_eq_ (1 − e^−kt^), where C_t_ is the concentration at time t, C_eq_ is the predicted equilibrium concentration, and k is the rate constant. The horizontal dotted line denotes the predicted C_eq_ for each plastic type. Where no equation is given, the model could not be fit to the data and where no horizontal line is given the non-linear regression was not statistically significant (p>0.05).

**Figure 4:**
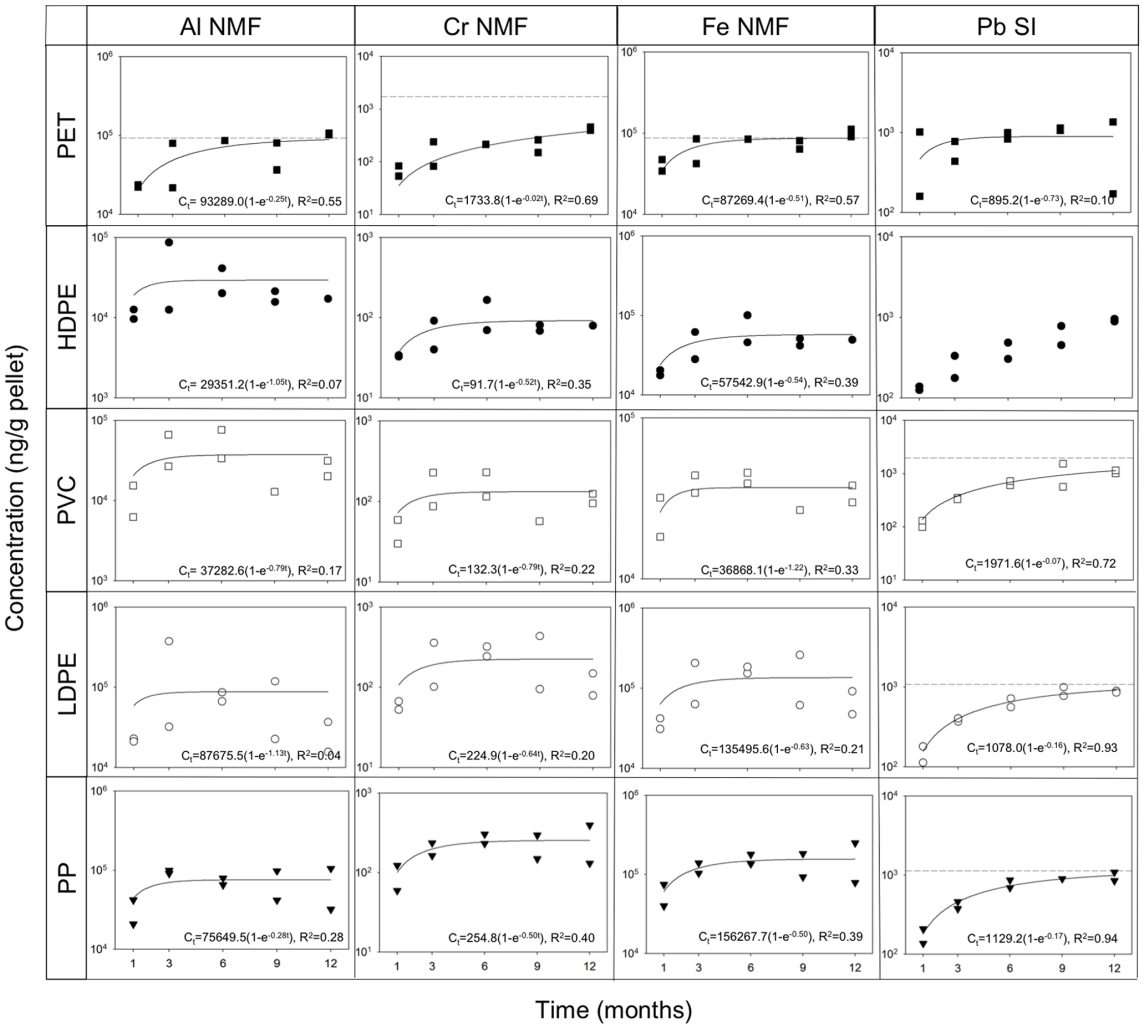
Concentrations of Al, Cr, Fe and Pb over time. Concentrations of Al, Cr, Fe and Pb (ng/g of pellets) vs time for each type of plastic at Nimitz Marine Facility (NMF) or Shelter Island (SI) where contamination was greatest. Rows represent plastic types PET, HDPE, PVC, LDPE and PP (in order from top to bottom). Columns represent metals ordered from left to right according to molecular weight. Note that vertical axes differ among graphs. Data were fit to the first-order approach to equilibrium model [26] using the exponential rise to maximum equation C_t_  =  C_eq_ (1 − e^−kt^), where C_t_ is the concentration at time t, C_eq_ is the predicted equilibrium concentration, and k is the rate constant. The horizontal dotted line denotes the predicted C_eq_ for each plastic type. Where no equation is given, the model could not be fit to the data and where no horizontal line is given the non-linear regression was not statistically significant (p>0.05).

We quantified temporal patterns of metal accumulation by each of the five plastic types at each of the three locations in San Diego Bay (Figures 3, 4 and S11 – S19). Fitting the first-order approach to equilibrium model [34] assumes a relatively constant background concentration of metals. Although this assumption likely does not hold for chemical pollutants during field deployment, the equation fits our data reasonably well for several metals over the long time scales of our experiment. Where the first-order approach to equilibrium model could be fit to the data and the non-linear regression was statistically significant (p<0.05), concentrations of all metals on all plastic types continued to increase after the 1-month sampling.

For Ni, Zn, Pb, Cr, Mn and Co predicted equilibrium concentrations (C_eq_) were not achieved for some polymers on either replicate sample during the entire 12-month period at several locations (Figures 3, 4 and S11 – S19). The US EPA Clean Water Act currently lists three of these metals, Ni, Zn and Pb, as priority pollutants. Concentrations of Ni did not reach equilibrium on PET at Coronado Cays (Figure 3). Concentrations of Zn did not reach equilibrium on PP at Nimitz Marine Facility (Figure S17). Concentrations of Pb did not reach equilibrium on PET and HDPE at Coronado Cays, on PVC at Coronado Cays and Shelter Island, on LDPE at Shelter Island and on PP at Shelter Island and Nimitz Marine Facility (Figures 4 and S19). Concentrations of Cr did not reach equilibrium on PET at Nimitz Marine Facility (Figures 4 and S12). Concentrations of Mn did not reach equilibrium on PET and PVC at Coronado Cays and on LDPE at Nimitz Marine Facility (Figures 3 and S13). Concentrations of Co did not reach equilibrium on PVC, LDPE and PP at Nimitz Marine Facility (Figure S15).

## Discussion

Overall, our work shows that a complex mixture of metals is found on plastic debris composed of the five most commonly produced types of plastic [30]. Our results suggest that the accumulation of metals on plastic debris may not differ greatly by polymer type as they do for organic chemical pollutants [13]. Here, patterns among polymers generally varied based on the specific metal, location and sampling period. Plastic type appears to be less important for the accumulation of metals than it is for organic contaminants. A possible explanation is that the accumulation of metals to plastic may be mediated by a biofilm. In the aquatic environment, including marine systems, it is well established that biofilms have sorptive properties and accumulate metals and other contaminants [35]. As such, biofilms have been used in rivers as passive samplers for metals [36]. It has been suggested that the composition of biofilm does not vary significantly among plastic types [24], [25], which may indicate that effects from the biofilm might overwhelm any differences in metal accumulation related to different types of plastic. While our objectives and analyses were not aimed at addressing the extent to which accumulation is mediated by a biofilm, which is immaterial in terms of risk, future research in the laboratory should test this hypothesis. Still, HDPE tended to accumulate lesser concentrations of metals—accumulating significantly less Cr, Ni, Zn, Cd and Pb than the other four plastic-types at several locations and sampling periods (Table S1). Four of these metals (Ni, Zn, Cd and Pb) are listed as priority pollutants by the United States and three (Ni, Cd, Pb) as priority substances by the European Union. This suggests that HDPE plastic debris may contain lesser concentrations of metals listed as priority pollutants, potentially bioavailable to aquatic animals, than other plastic debris. This contrasts with our previous work showing that HDPE accumulates greater concentrations of PBTs [13]. Thus, when assessing the hazards associated with aquatic plastic debris it is important to think holistically about which chemical pollutants the plastic is exposed to in the environment.

Plastic will be exposed to different mixtures and concentrations of chemical pollutants based upon the location where it has been discarded [12], [13]. In San Diego Bay, metal contamination is a problem, originating from multiple recurring past and present sources mainly linked to military and recreational boating (e.g. antifouling, paint, fuel combustion and shipyard activity) [37]. We found that the concentration of each metal on plastic pellets varied among locations. Metal concentrations at Nimitz Marine Facility and Shelter Island (in the northern part of the bay) are generally indistinguishable and significantly different from Coronado Cays (in the southern part of the bay). Nimitz Marine Facility and Shelter Island are near the mouth of the bay and are flushed to a grater extent than Coronado Cays, which is in the back of the bay [37]. Fe, Pb, Cr and Al concentrations were generally greater on pellets deployed at Nimitz Marine Facility and/or Shelter Island. In contrast, Cd, Zn, Ni, Mn and Co concentrations were generally greater on pellets deployed at Coronado Cays. These differences are expected based upon their local sources to the bay (e.g., stormwater runoff, shipyard activity, recreational boating) and prior research showing that concentrations of sorbed pollutants on plastic reflect regional differences [13]–[15], [17]. Spatial variation among concentrations and mixtures of sorbed pollutants on plastic has implications for management. Plastic debris discarded in locations where contamination is greater and from diverse sources may be a greater management priority because it may be associated with a more complex mixture of large concentrations of contaminants, which may pose a greater hazard to aquatic animals that ingest the debris.

Similarly, the length of time plastic remains aquatic debris has implications for management. Our previous work shows that plastic pellets accumulate greater concentrations of organic contaminants the longer they remain at sea [13]. Similarly, in this study we found that metal concentrations increase over time and reach equilibrium on plastic pellets much slower in the aquatic environment than observed in the laboratory [15], [20]. In the laboratory, Co, Cr, Ni and Pb sorbed to polyethylene (PE) pellets reached equilibrium concentrations in less than 100 hours [15]. Our analyses of HDPE and LDPE pellets in the northern part of the bay shows that concentrations of Co and Pb did not reach equilibrium during the entire 12-months, concentrations of Cr did not reach equilibrium during the first 9 months, and concentrations of Ni did not reach equilibrium during the first 3 months. Using the predicted concentration at equilibrium (C_eq_), and the assumption that metal accumulation patterns will follow the exponential rise to maximum equation, Co is predicted to reach equilibrium on polyethylene at 22 months and Pb at 64 months. Comparing plastic debris to plastic in a controlled laboratory setting has limited applicability to quantifying chemical hazards associated with plastic debris in aquatic habitats. Concentrations of ambient metals will change over time based upon their sources and physical changes in the water column (e.g. temperature). As plastic debris weathers it will gain surface area, generate oxygen groups (increasing polarity) [26], [38] and foul (increasing their charge, roughness and porosity) [39]—all enhancing the reactivity of the surface [15]. Over time, as biofilms continuously accrue to the surface of plastic, the accumulation of metals is likely continually enhanced [23] [36]. In fact, in the laboratory, aged pellets have been found to have a greater capacity for metals than virgin pellets [15]. Plastic debris remains in the environment for a long period of time and sorbs increasing concentrations of a mixture of pollutants over time [13], [38]. Our results further confirm that plastic accumulates greater concentrations of chemical contaminants, and thus may become more hazardous to animals if ingested, the longer it remains in the aquatic environment.

It is well-known that a wide range of animals ingest plastic debris in nature, including invertebrates [40], fish [41], [42], marine mammals [5], [43], seabirds [5], [44] and sea turtles [5], [45]. Because metals on pellets accumulate on the surface or are associated with hydrogenous or biogenic phases, they are potentially bioavailable [15]. Strikingly, we found that concentrations of some metals on plastic pellets are similar or greater than those found by other researchers on natural particulates in San Diego Bay at similar locations and during similar seasons. For example, concentrations of Fe, Mn and Zn were two orders of magnitude greater and Cd within an order of magnitude on plastic pellets than in seawater particulates [37] and concentrations of Al and Fe were an order of magnitude greater and Mn within an order of magnitude on plastic pellets than in sediment [46] samples. This suggests that some metals may partition to plastic debris to a greater extent than to natural sediment. Future laboratory studies should test this hypothesis as it implies that plastic debris may pose a similar or greater threat of contributing to the bioaccumulation of metals relative to natural particulates.

Exposure to metals may cause ecological effects from decreased growth and survival [47], [48] to decreases in biodiversity [49], [50]. While the toxicity of metals may be well-studied, the bioavailability and ecological risks of metal contaminants on plastics remain unknown and deserving of future examination. The bioavailability of metals is influenced via the route of exposure (e.g., ingestion of plastic debris) and health effects will differ according to the mixture of metals and the species exposed [51]. Moreover, the hazards associated with the complex mixture of metals, plastic and organic pollutants require attention. Future research efforts assessing the hazards associated with the exposure of plastic debris to wildlife should consider the polymer type, location of debris and the length of time the debris has persisted in the environment—all affecting the complex mixture of sorbed pollutants (e.g., PBTs, metals) and chemical ingredients (e.g., vinyl chloride, styrene, phthalates) associated with plastic debris.

## Supporting Information

Figure S1 **Schematic diagram of the experimental design at each location.** Two replicate samples of each plastic type (HDPE, LDPE, PP, PET and PVC) were deployed for future collection at the end of five time periods: 1, 3, 6, 9, and 12 months (50 samples per location, 25 samples per PVC pipe and 150 total samples across all 3 locations). Each replicate sample consisted of 5 g of pellets of one plastic type placed in individual Nitex mesh (1.3 mm) bags placed inside a nylon mesh (10 mm) bag. Replicate samples were deployed by hanging each nylon bag on one of two identical PVC frames suspended from each dock. On each dock, the two PVC frames were positioned approximately 2 meters from each other. Bags containing plastic samples were suspended at a depth approximately 0.5 m below the surface of floating docks or 0.5 m below Mean Lower Low Water at Nimitz Marine Facility.
(TIFF)

Figure S2 **Al concentrations among polymers.** Total Al concentration for each of five plastic types (PET-black, HDPE-hatched, PVC-diagonal stripes, LDPE-white, PP-horizontal stripes) at each of three sites (CC-Cornado Cays, SI-Shelter Island, NMF-Nimitz Marine Facility) for the 1-, 3-, 6-, 9- and 12-month sampling periods ordered from top to bottom from the shortest to the longest deployment. Each bar represents the mean concentration (ng/g + S.E.; n = 2). Where SNK tests showed significantly different treatment means (*P*<0.05), the same letter coding represents groups that are statistically similar at a given location.
(TIFF)

Figure S3 **Cr concentrations among polymers.** Total Cr concentration for each of five plastic types (PET-black, HDPE-hatched, PVC-diagonal stripes, LDPE-white, PP-horizontal stripes) at each of three sites (CC-Cornado Cays, SI-Shelter Island, NMF-Nimitz Marine Facility) for the 1-, 3-, 6-, 9- and 12-month sampling periods ordered from top to bottom from the shortest to the longest deployment. Each bar represents the mean concentration (ng/g + S.E.; n = 2).
(TIFF)

Figure S4 **Mn concentrations among polymers.** Total Mn concentration for each of five plastic types (PET-black, HDPE-hatched, PVC-diagonal stripes, LDPE-white, PP-horizontal stripes) at each of three sites (CC-Cornado Cays, SI-Shelter Island, NMF-Nimitz Marine Facility) for the 1-, 3-, 6-, 9- and 12-month sampling periods ordered from top to bottom from the shortest to the longest deployment. Each bar represents the mean concentration (ng/g + S.E.; n = 2).
(TIFF)

Figure S5 **Fe concentrations among polymers.** Total Fe concentration for each of five plastic types (PET-black, HDPE-hatched, PVC-diagonal stripes, LDPE-white, PP-horizontal stripes) at each of three sites (CC-Cornado Cays, SI-Shelter Island, NMF-Nimitz Marine Facility) for the 1-, 3-, 6-, 9- and 12-month sampling periods ordered from top to bottom from the shortest to the longest deployment. Each bar represents the mean concentration (ng/g + S.E.; n = 2). Where SNK tests showed significantly different treatment means (*P*<0.05), the same letter coding represents groups that are statistically similar at a given location.
(TIFF)

Figure S6 **Co concentrations among polymers.** Total Co concentration for each of five plastic types (PET-black, HDPE-hatched, PVC-diagonal stripes, LDPE-white, PP-horizontal stripes) at each of three sites (CC-Cornado Cays, SI-Shelter Island, NMF-Nimitz Marine Facility) for the 1-, 3-, 6-, 9- and 12-month sampling periods ordered from top to bottom from the shortest to the longest deployment. Each bar represents the mean concentration (ng/g + S.E.; n = 2).
(TIFF)

Figure S7 **Ni concentrations among polymers.** Total Ni concentration for each of five plastic types (PET-black, HDPE-hatched, PVC-diagonal stripes, LDPE-white, PP-horizontal stripes) at each of three sites (CC-Cornado Cays, SI-Shelter Island, NMF-Nimitz Marine Facility) for the 1-, 3-, 6-, 9- and 12-month sampling periods ordered from top to bottom from the shortest to the longest deployment. Each bar represents the mean concentration (ng/g + S.E.; n = 2). A non-detect is denoted by nd. Where SNK tests showed significantly different treatment means (*P*<0.05), the same letter coding represents groups that are statistically similar at a given location.
(TIFF)

Figure S8 **Zn concentrations among polymers.** Total Zn concentration for each of five plastic types (PET-black, HDPE-hatched, PVC-diagonal stripes, LDPE-white, PP-horizontal stripes) at each of three sites (CC-Cornado Cays, SI-Shelter Island, NMF-Nimitz Marine Facility) for the 1-, 3-, 6-, 9- and 12-month sampling periods ordered from top to bottom from the shortest to the longest deployment. Each bar represents the mean concentration (ng/g + S.E.; n = 2). Where SNK tests showed significantly different treatment means (*P*<0.05), the same letter coding represents groups that are statistically similar at a given location.
(TIFF)

Figure S9 **Cd concentrations among polymers.** Total Cd concentration for each of five plastic types (PET-black, HDPE-hatched, PVC-diagonal stripes, LDPE-white, PP-horizontal stripes) at each of three sites (CC-Cornado Cays, SI-Shelter Island, NMF-Nimitz Marine Facility) for the 1-, 3-, 6-, 9- and 12-month sampling periods ordered from top to bottom from the shortest to the longest deployment. Each bar represents the mean concentration (ng/g + S.E.; n = 2). A non-detect is denoted by nd. Where SNK tests showed significantly different treatment means (*P*<0.05), the same letter coding represents groups that are statistically similar at a given location.
(TIFF)

Figure S10 **Pb concentrations among polymers.** Total Pb concentration for each of five plastic types (PET-black, HDPE-hatched, PVC-diagonal stripes, LDPE-white, PP-horizontal stripes) at each of three sites (CC-Cornado Cays, SI-Shelter Island, NMF-Nimitz Marine Facility) for the 1-, 3-, 6-, 9- and 12-month sampling periods ordered from top to bottom from the shortest to the longest deployment. Each bar represents the mean concentration (ng/g + S.E.; n = 2). Where SNK tests showed significantly different treatment means (*P*<0.05), the same letter coding represents groups that are statistically similar at a given location.
(TIFF)

Figure S11 **Concentrations of Al over time.** Concentration of Al (ng/g of pellets) vs. time for each type of plastic (rows) at Coronado Cays (CC; left), and Shelter Island (SI; middle), and Nimitz Marine Facility (NMF; right). Note that vertical axes differ among graphs. Data were fit to the first-order approach to equilibrium model [26] using the exponential rise to maximum equation C_t_ = C_eq_(1-e^-kt^), where C_t_ is the concentration at time t, C_eq_ is the predicted equilibrium concentration, and k is the rate constant. The horizontal dotted line denotes the predicted C_eq_ for each plastic type. Where no equation is given, the model could not be fit to the data and where no horizontal line is given the non-linear regression was not statistically significant (p>0.05).
(TIFF)

Figure S12 **Concentrations of Cr over time.** Concentration of Cr (ng/g of pellets) vs. time for each type of plastic (rows) at Coronado Cays (CC; left), and Shelter Island (SI; middle), and Nimitz Marine Facility (NMF; right). Note that vertical axes differ among graphs. Data were fit to the first-order approach to equilibrium model [26] using the exponential rise to maximum equation C_t_ = C_eq_(1-e^-kt^), where C_t_ is the concentration at time t, C_eq_ is the predicted equilibrium concentration, and k is the rate constant. The horizontal dotted line denotes the predicted C_eq_ for each plastic type. Where no equation is given, the model could not be fit to the data and where no horizontal line is given the non-linear regression was not statistically significant (p>0.05).
(TIFF)

Figure S13 **Concentrations of Mn over time.** Concentration of Mn (ng/g of pellets) vs. time for each type of plastic (rows) at Coronado Cays (CC; left), and Shelter Island (SI; middle), and Nimitz Marine Facility (NMF; right). Note that vertical axes differ among graphs. Data were fit to the first-order approach to equilibrium model [26] using the exponential rise to maximum equation C_t_ = C_eq_(1-e^-kt^), where C_t_ is the concentration at time t, C_eq_ is the predicted equilibrium concentration, and k is the rate constant. The horizontal dotted line denotes the predicted C_eq_ for each plastic type. Where no equation is given, the model could not be fit to the data and where no horizontal line is given the non-linear regression was not statistically significant (p>0.05).
(TIFF)

Figure S14 **Concentrations of Fe over time.** Concentration of Fe (ng/g of pellets) vs. time for each type of plastic (rows) at Coronado Cays (CC; left), and Shelter Island (SI; middle), and Nimitz Marine Facility (NMF; right). Note that vertical axes differ among graphs. Data were fit to the first-order approach to equilibrium model [26] using the exponential rise to maximum equation C_t_ = C_eq_(1-e^-kt^), where C_t_ is the concentration at time t, C_eq_ is the predicted equilibrium concentration, and k is the rate constant. The horizontal dotted line denotes the predicted C_eq_ for each plastic type. Where no equation is given, the model could not be fit to the data and where no horizontal line is given the non-linear regression was not statistically significant (p>0.05).
(TIFF)

Figure S15 **Concentrations of Co over time.** Concentration of Co (ng/g of pellets) vs. time for each type of plastic (rows) at Coronado Cays (CC; left), and Shelter Island (SI; middle), and Nimitz Marine Facility (NMF; right). Note that vertical axes differ among graphs. Data were fit to the first-order approach to equilibrium model [26] using the exponential rise to maximum equation C_t_ = C_eq_(1-e^-kt^), where C_t_ is the concentration at time t, C_eq_ is the predicted equilibrium concentration, and k is the rate constant. The horizontal dotted line denotes the predicted C_eq_ for each plastic type. Where no equation is given, the model could not be fit to the data and where no horizontal line is given the non-linear regression was not statistically significant (p>0.05).
(TIFF)

Figure S16 **Concentrations of Ni over time.** Concentration of Ni (ng/g of pellets) vs. time for each type of plastic (rows) at Coronado Cays (CC; left), and Shelter Island (SI; middle), and Nimitz Marine Facility (NMF; right). Note that vertical axes differ among graphs. Data were fit to the first-order approach to equilibrium model [26] using the exponential rise to maximum equation C_t_ = C_eq_(1-e^-kt^), where C_t_ is the concentration at time t, C_eq_ is the predicted equilibrium concentration, and k is the rate constant. The horizontal dotted line denotes the predicted C_eq_ for each plastic type. Where no equation is given, the model could not be fit to the data and where no horizontal line is given the non-linear regression was not statistically significant (p>0.05).
(TIFF)

Figure S17 **Concentrations of Zn over time.** Concentration of Zn (ng/g of pellets) vs. time for each type of plastic (rows) at Coronado Cays (CC; left), and Shelter Island (SI; middle), and Nimitz Marine Facility (NMF; right). Note that vertical axes differ among graphs. Data were fit to the first-order approach to equilibrium model [26] using the exponential rise to maximum equation C_t_ = C_eq_(1-e^-kt^), where C_t_ is the concentration at time t, C_eq_ is the predicted equilibrium concentration, and k is the rate constant. The horizontal dotted line denotes the predicted C_eq_ for each plastic type. Where no equation is given, the model could not be fit to the data and where no horizontal line is given the non-linear regression was not statistically significant (p>0.05).
(TIFF)

Figure S18 **Concentrations of Cd over time.** Concentration of Cd (ng/g of pellets) vs. time for each type of plastic (rows) at Coronado Cays (CC; left), and Shelter Island (SI; middle), and Nimitz Marine Facility (NMF; right). Note that vertical axes differ among graphs. Data were fit to the first-order approach to equilibrium model [26] using the exponential rise to maximum equation C_t_ = C_eq_(1-e^-kt^), where C_t_ is the concentration at time t, C_eq_ is the predicted equilibrium concentration, and k is the rate constant. The horizontal dotted line denotes the predicted C_eq_ for each plastic type. Where no equation is given, the model could not be fit to the data and where no horizontal line is given the non-linear regression was not statistically significant (p>0.05).
(TIFF)

Figure S19 **Concentrations of Pb over time.** Concentration of Pb (ng/g of pellets) vs. time for each type of plastic (rows) at Coronado Cays (CC; left), and Shelter Island (SI; middle), and Nimitz Marine Facility (NMF; right). Note that vertical axes differ among graphs. Data were fit to the first-order approach to equilibrium model [26] using the exponential rise to maximum equation C_t_ = C_eq_(1-e^-kt^), where C_t_ is the concentration at time t, C_eq_ is the predicted equilibrium concentration, and k is the rate constant. The horizontal dotted line denotes the predicted C_eq_ for each plastic type. Where no equation is given, the model could not be fit to the data and where no horizontal line is given the non-linear regression was not statistically significant (p>0.05).
(TIFF)

Table S1 **Concentrations of all terageted metals on individual samples for each time period among all plastic types at all three locations.** Each individual table shows data for each of the five time periods. Concentrations of metals are given in ng/g pellet.
(PDF)

Table S2 **Two-way Analysis of Variance (ANOVA) tables for each metal individually (in order of molecular weight) between polymers and locations within San Diego Bay for each of the five time periods (1, 3, 6, 9 and 12 months).** SNK results are given in order from highest to lowest concentration for each contaminant group among polymers and locations.
(PDF)
