# Supplementary figures and images for: Long-Term Sorption of Metals Is Similar among Plastic Types: Implications for Plastic Debris in Aquatic Environments
Source: PLoS One. 2014 Jan 15;9(1):e85433. doi: 10.1371/journal.pone.0085433 (PMC3893203; doi:10.1371/journal.pone.0085433)

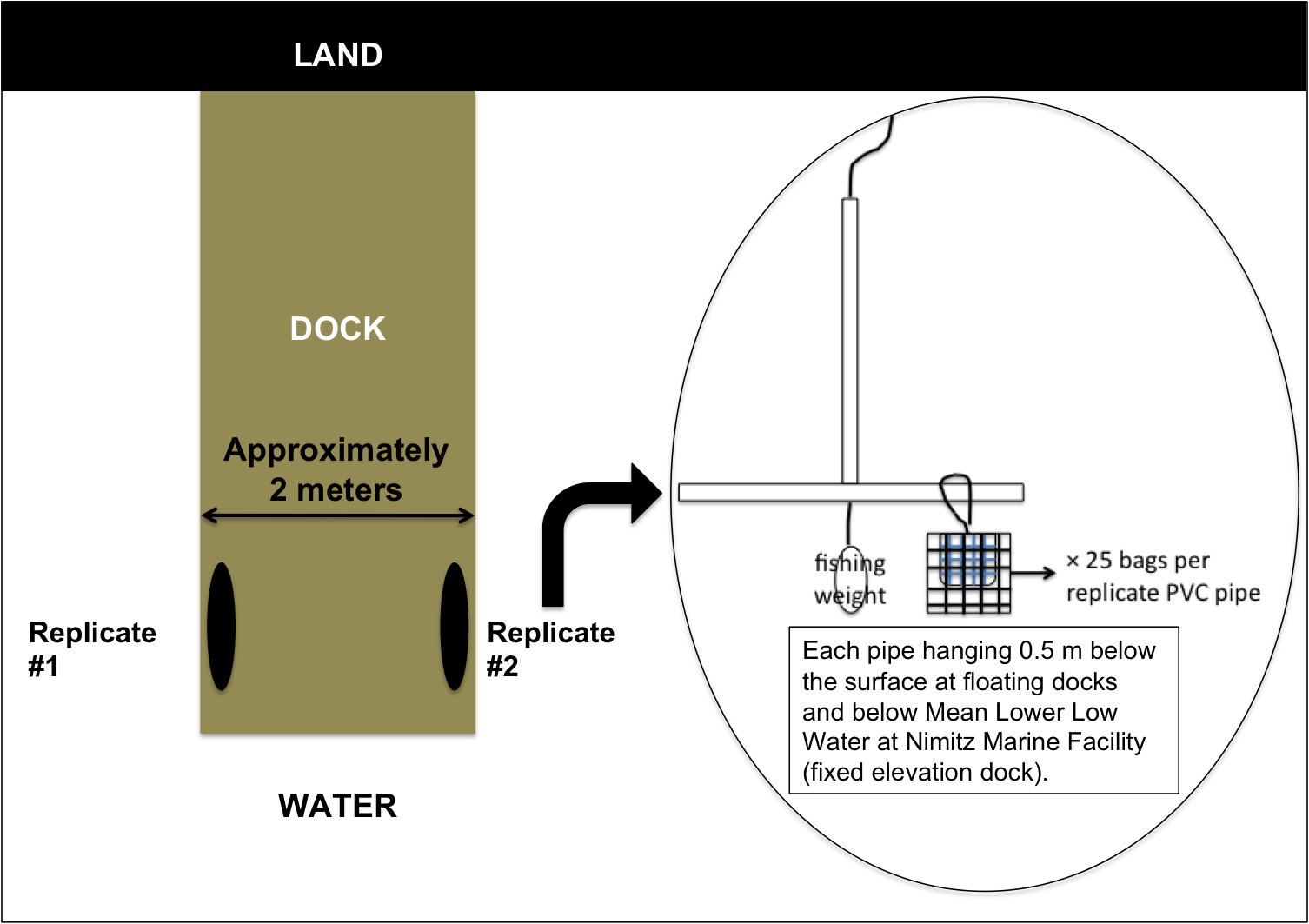

Supplement: Figure S1 — Schematic diagram of the experimental design at each location. Two replicate samples of each plastic type (HDPE, LDPE, PP, PET and PVC) were deployed for future collection at the end of five time periods: 1, 3, 6, 9, and 12 months (50 samples per location, 25 samples per PVC pipe and 150 total samples across all 3 locations). Each replicate sample consisted of 5 g of pellets of one plastic type placed in individual Nitex mesh (1.3 mm) bags placed inside a nylon mesh (10 mm) bag. Replicate samples were deployed by hanging each nylon bag on one of two identical PVC frames suspended from each dock. On each dock, the two PVC frames were positioned approximately 2 meters from each other. Bags containing plastic samples were suspended at a depth approximately 0.5 m below the surface of floating docks or 0.5 m below Mean Lower Low Water at Nimitz Marine Facility. (TIFF) [file pone.0085433.s001.tiff]

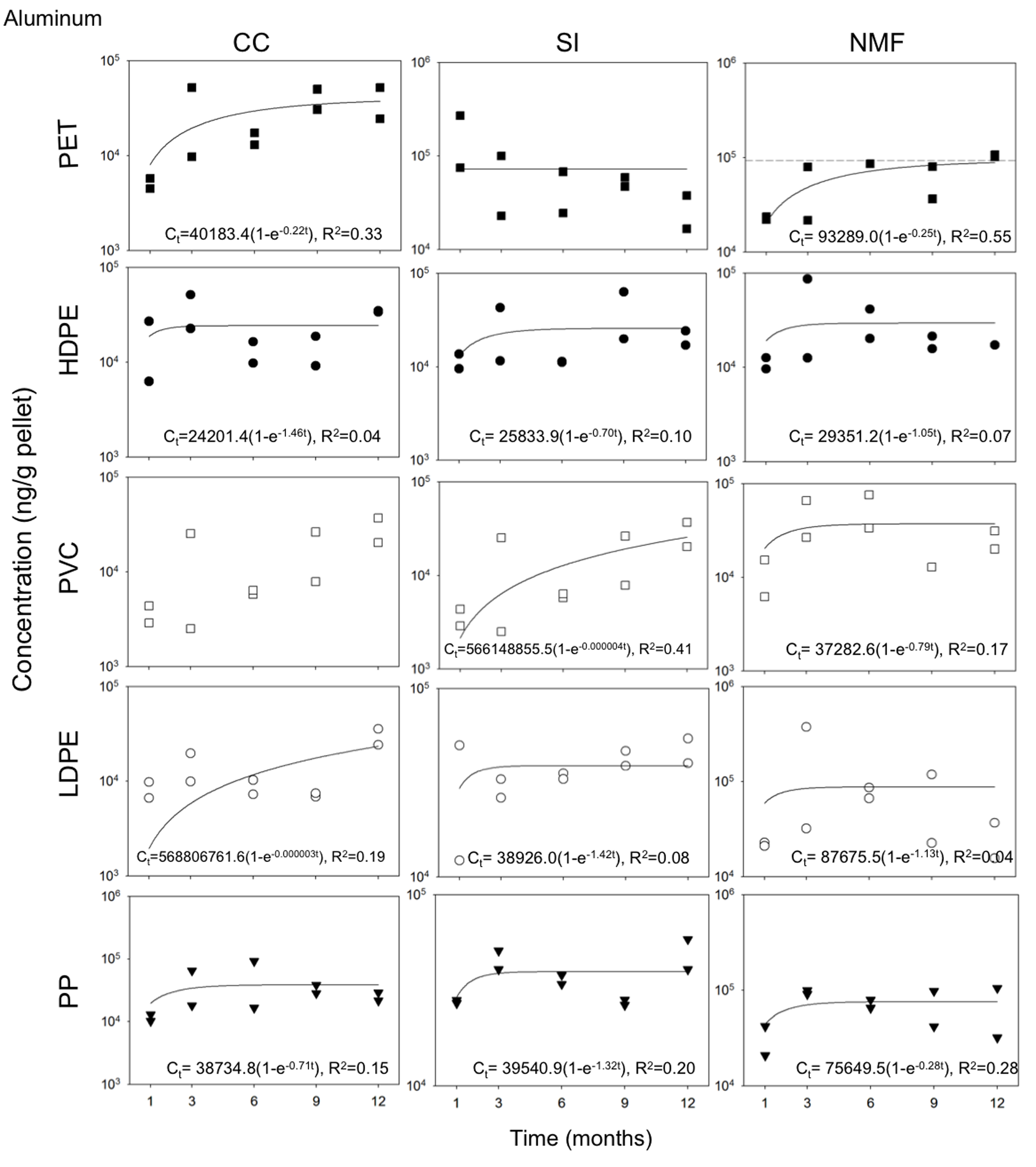

Supplement: Figure S11 — Concentrations of Al over time. Concentration of Al (ng/g of pellets) vs. time for each type of plastic (rows) at Coronado Cays (CC; left), and Shelter Island (SI; middle), and Nimitz Marine Facility (NMF; right). Note that vertical axes differ among graphs. Data were fit to the first-order approach to equilibrium model [26] using the exponential rise to maximum equation Ct = Ceq(1-e-kt), where Ct is the concentration at time t, Ceq is the predicted equilibrium concentration, and k is the rate constant. The horizontal dotted line denotes the predicted Ceq for each plastic type. Where no equation is given, the model could not be fit to the data and where no horizontal line is given the non-linear regression was not statistically significant (p>0.05). (TIFF) [file pone.0085433.s011.tiff]

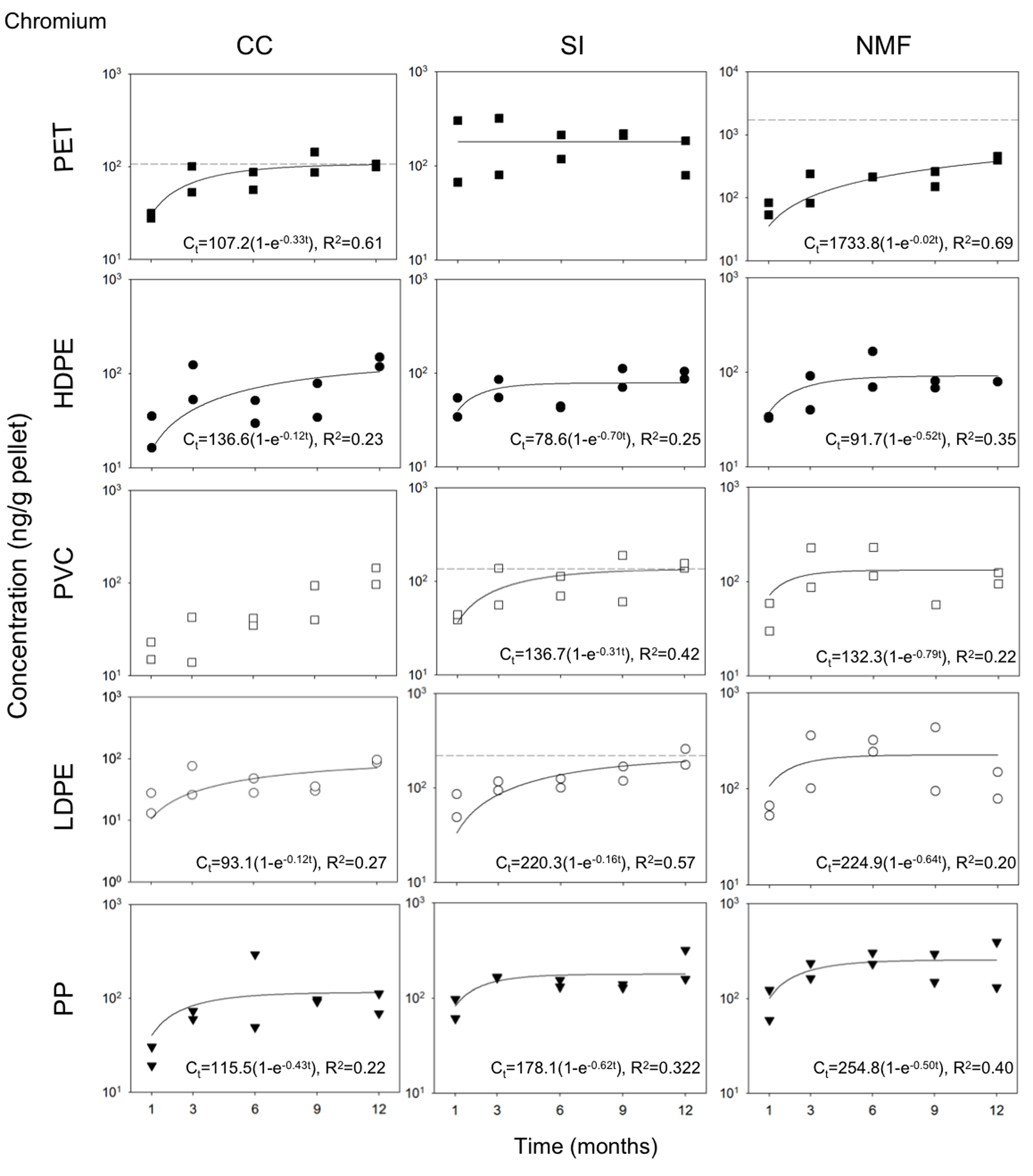

Supplement: Figure S12 — Concentrations of Cr over time. Concentration of Cr (ng/g of pellets) vs. time for each type of plastic (rows) at Coronado Cays (CC; left), and Shelter Island (SI; middle), and Nimitz Marine Facility (NMF; right). Note that vertical axes differ among graphs. Data were fit to the first-order approach to equilibrium model [26] using the exponential rise to maximum equation Ct = Ceq(1-e-kt), where Ct is the concentration at time t, Ceq is the predicted equilibrium concentration, and k is the rate constant. The horizontal dotted line denotes the predicted Ceq for each plastic type. Where no equation is given, the model could not be fit to the data and where no horizontal line is given the non-linear regression was not statistically significant (p>0.05). (TIFF) [file pone.0085433.s012.tiff]

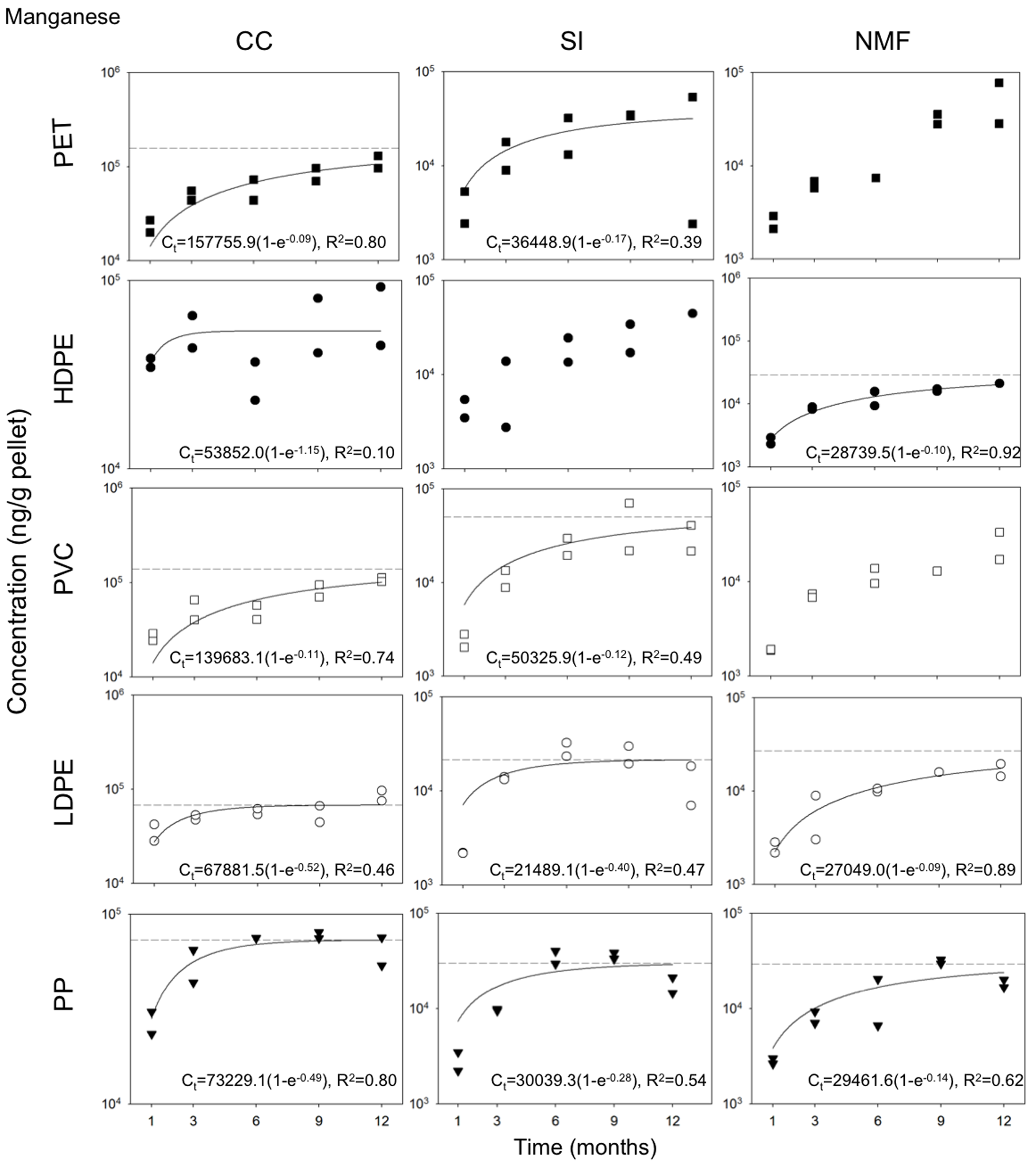

Supplement: Figure S13 — Concentrations of Mn over time. Concentration of Mn (ng/g of pellets) vs. time for each type of plastic (rows) at Coronado Cays (CC; left), and Shelter Island (SI; middle), and Nimitz Marine Facility (NMF; right). Note that vertical axes differ among graphs. Data were fit to the first-order approach to equilibrium model [26] using the exponential rise to maximum equation Ct = Ceq(1-e-kt), where Ct is the concentration at time t, Ceq is the predicted equilibrium concentration, and k is the rate constant. The horizontal dotted line denotes the predicted Ceq for each plastic type. Where no equation is given, the model could not be fit to the data and where no horizontal line is given the non-linear regression was not statistically significant (p>0.05). (TIFF) [file pone.0085433.s013.tiff]

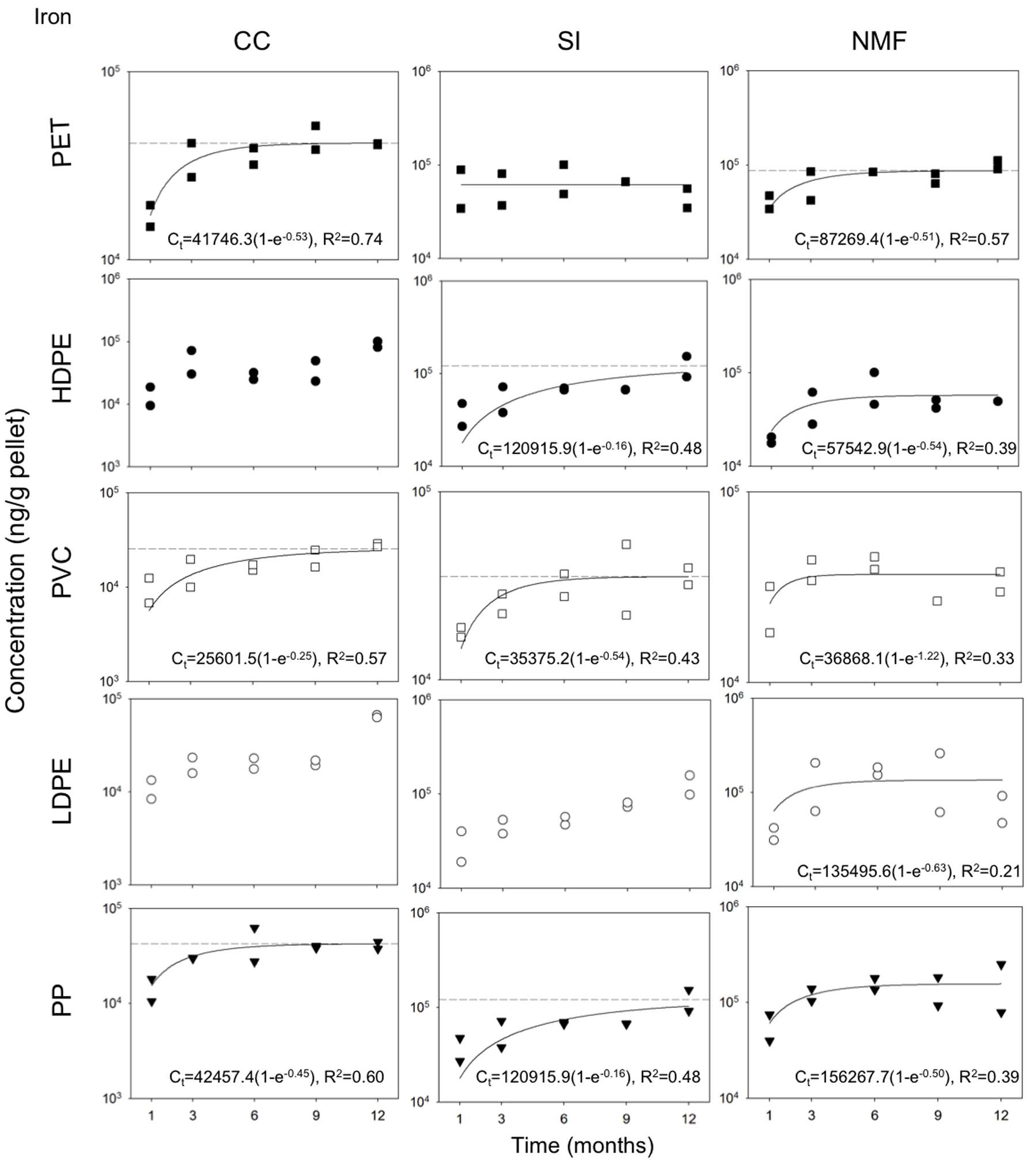

Supplement: Figure S14 — Concentrations of Fe over time. Concentration of Fe (ng/g of pellets) vs. time for each type of plastic (rows) at Coronado Cays (CC; left), and Shelter Island (SI; middle), and Nimitz Marine Facility (NMF; right). Note that vertical axes differ among graphs. Data were fit to the first-order approach to equilibrium model [26] using the exponential rise to maximum equation Ct = Ceq(1-e-kt), where Ct is the concentration at time t, Ceq is the predicted equilibrium concentration, and k is the rate constant. The horizontal dotted line denotes the predicted Ceq for each plastic type. Where no equation is given, the model could not be fit to the data and where no horizontal line is given the non-linear regression was not statistically significant (p>0.05). (TIFF) [file pone.0085433.s014.tiff]

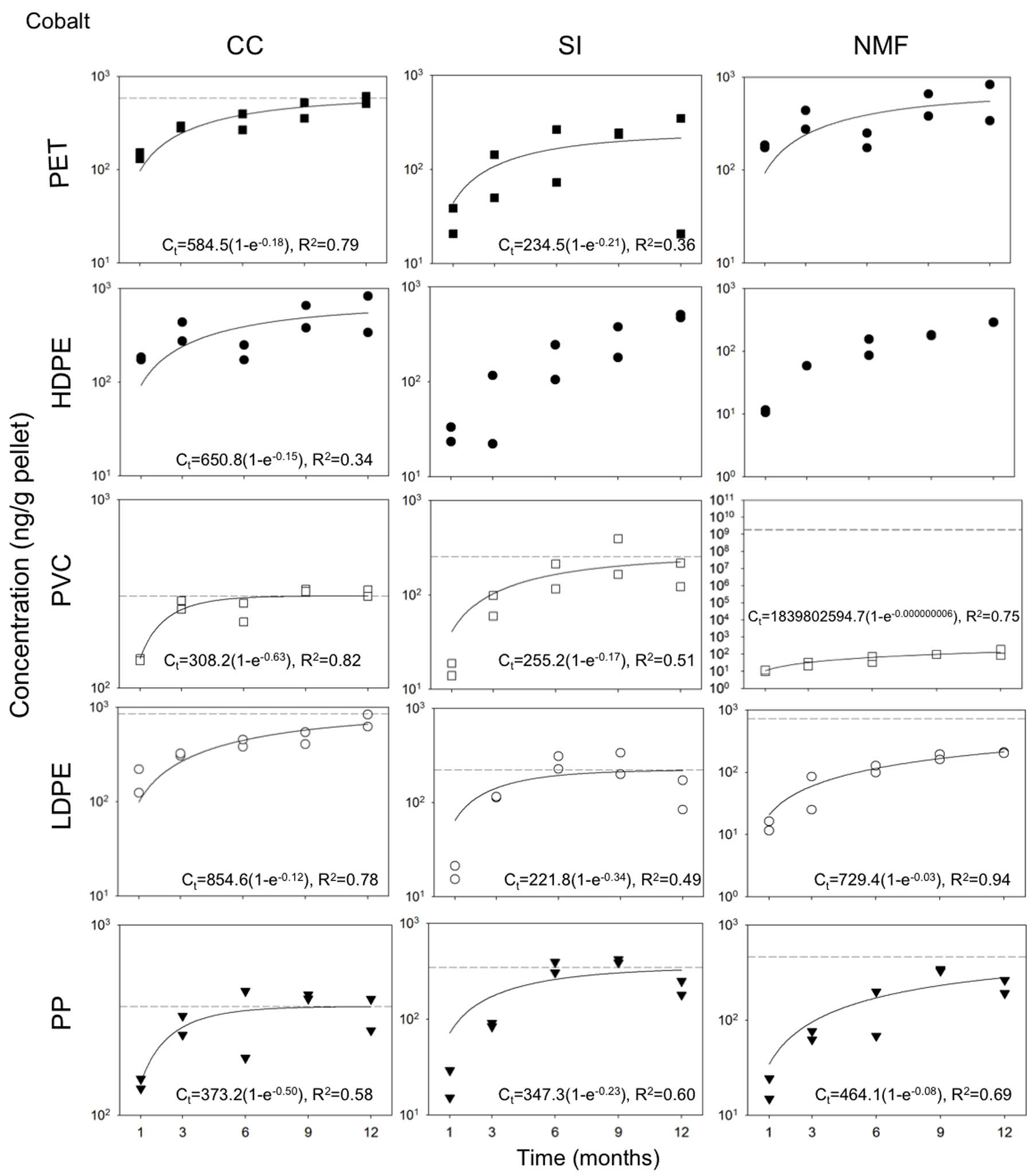

Supplement: Figure S15 — Concentrations of Co over time. Concentration of Co (ng/g of pellets) vs. time for each type of plastic (rows) at Coronado Cays (CC; left), and Shelter Island (SI; middle), and Nimitz Marine Facility (NMF; right). Note that vertical axes differ among graphs. Data were fit to the first-order approach to equilibrium model [26] using the exponential rise to maximum equation Ct = Ceq(1-e-kt), where Ct is the concentration at time t, Ceq is the predicted equilibrium concentration, and k is the rate constant. The horizontal dotted line denotes the predicted Ceq for each plastic type. Where no equation is given, the model could not be fit to the data and where no horizontal line is given the non-linear regression was not statistically significant (p>0.05). (TIFF) [file pone.0085433.s015.tiff]

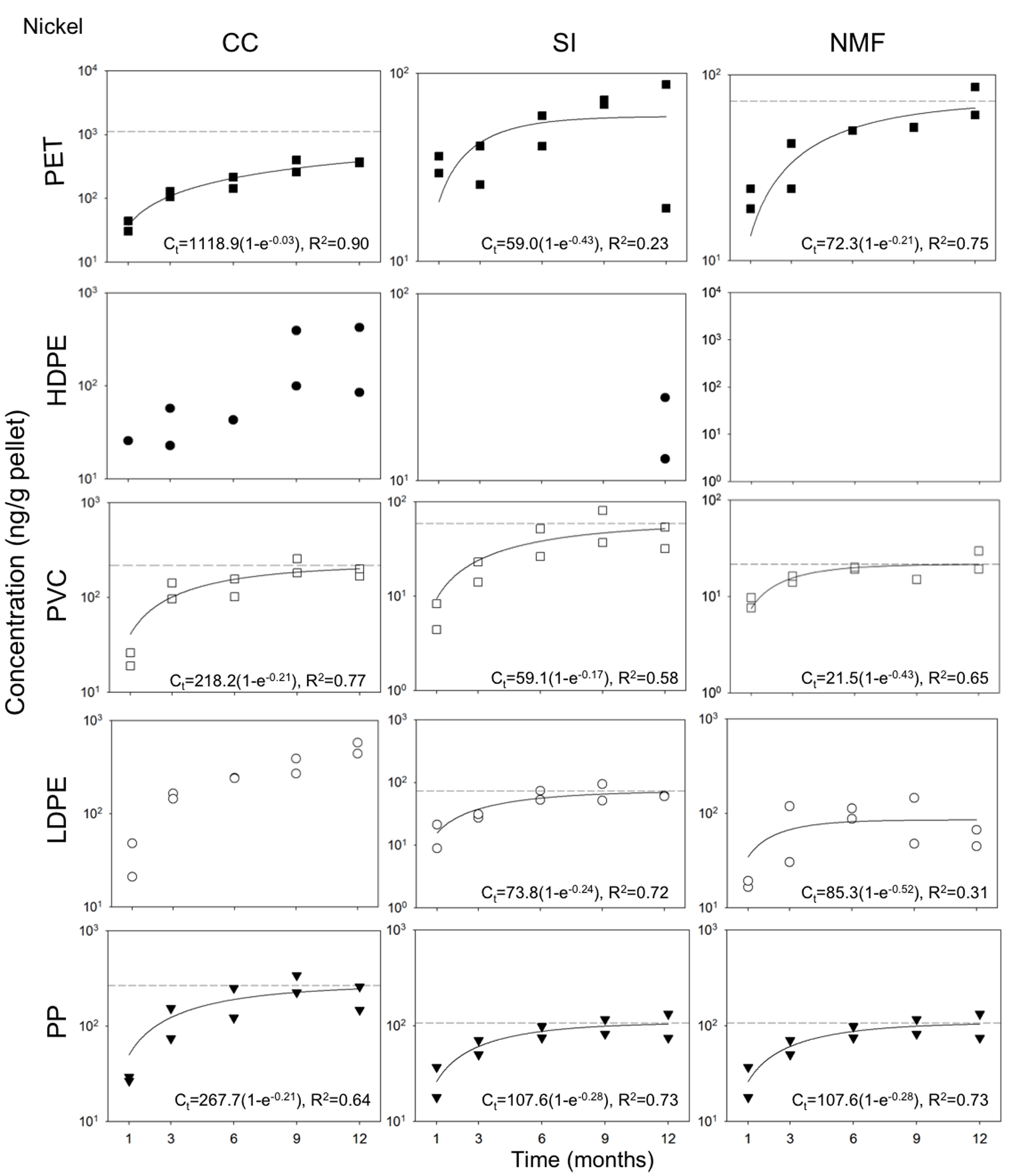

Supplement: Figure S16 — Concentrations of Ni over time. Concentration of Ni (ng/g of pellets) vs. time for each type of plastic (rows) at Coronado Cays (CC; left), and Shelter Island (SI; middle), and Nimitz Marine Facility (NMF; right). Note that vertical axes differ among graphs. Data were fit to the first-order approach to equilibrium model [26] using the exponential rise to maximum equation Ct = Ceq(1-e-kt), where Ct is the concentration at time t, Ceq is the predicted equilibrium concentration, and k is the rate constant. The horizontal dotted line denotes the predicted Ceq for each plastic type. Where no equation is given, the model could not be fit to the data and where no horizontal line is given the non-linear regression was not statistically significant (p>0.05). (TIFF) [file pone.0085433.s016.tiff]

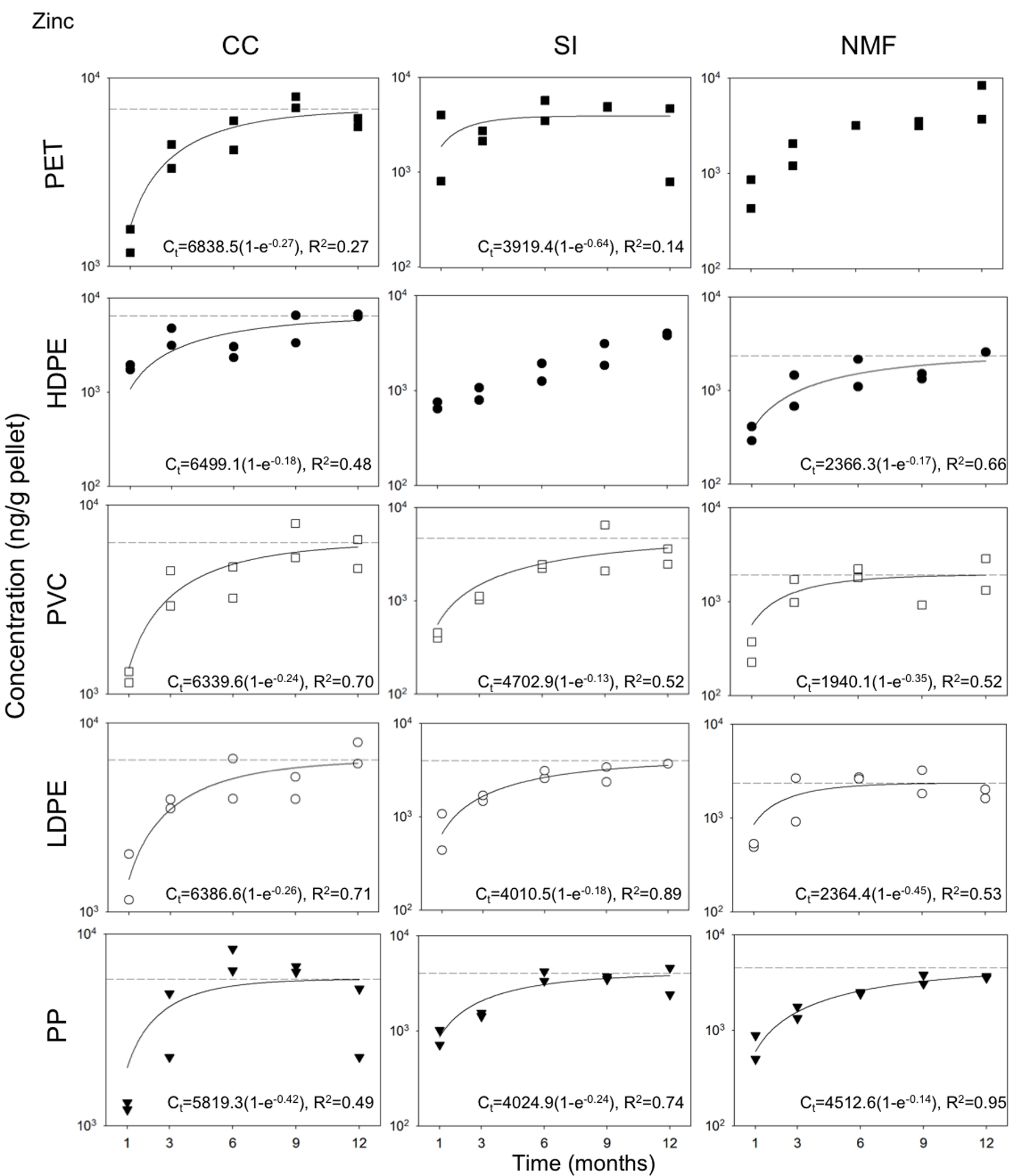

Supplement: Figure S17 — Concentrations of Zn over time. Concentration of Zn (ng/g of pellets) vs. time for each type of plastic (rows) at Coronado Cays (CC; left), and Shelter Island (SI; middle), and Nimitz Marine Facility (NMF; right). Note that vertical axes differ among graphs. Data were fit to the first-order approach to equilibrium model [26] using the exponential rise to maximum equation Ct = Ceq(1-e-kt), where Ct is the concentration at time t, Ceq is the predicted equilibrium concentration, and k is the rate constant. The horizontal dotted line denotes the predicted Ceq for each plastic type. Where no equation is given, the model could not be fit to the data and where no horizontal line is given the non-linear regression was not statistically significant (p>0.05). (TIFF) [file pone.0085433.s017.tiff]

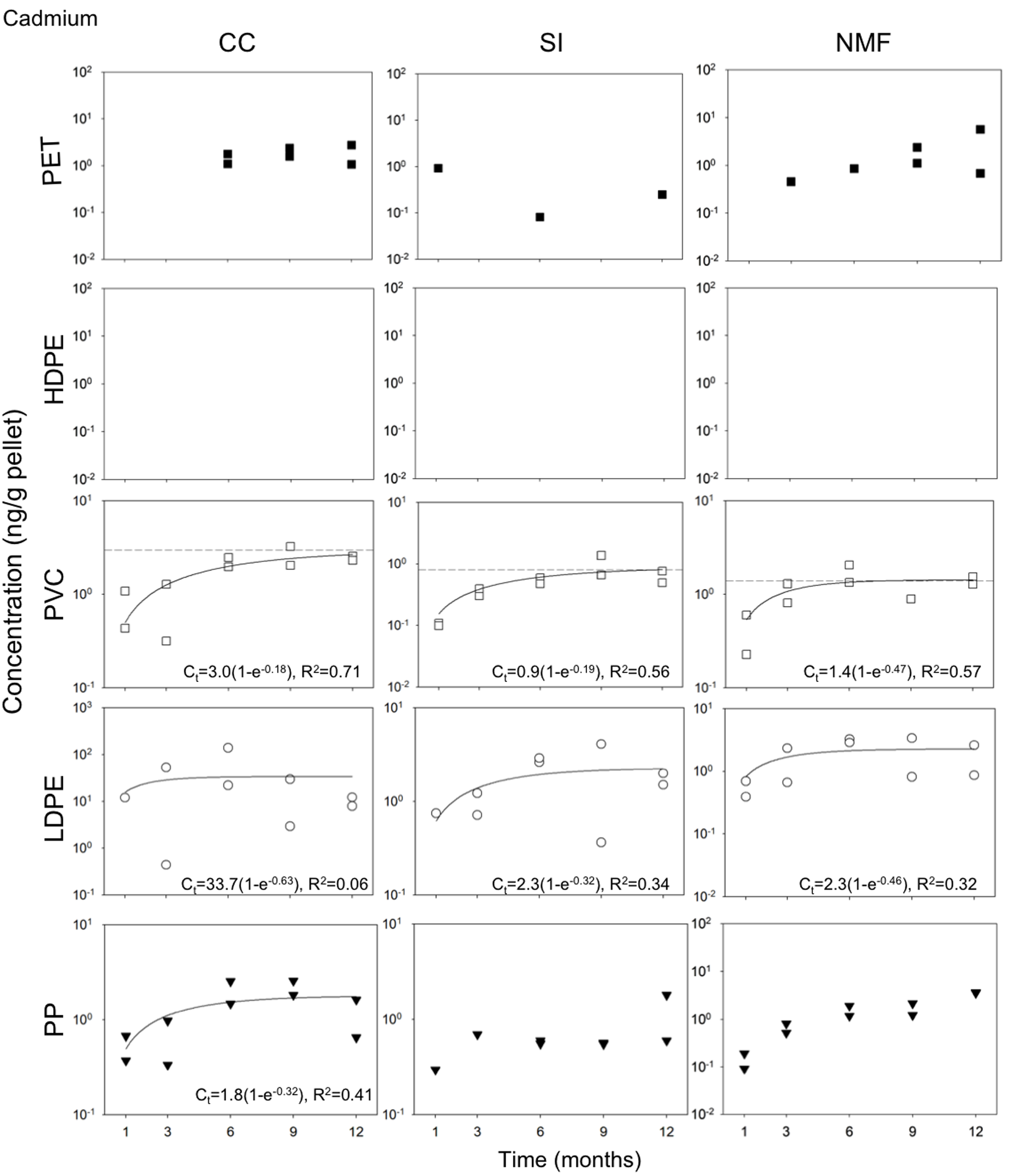

Supplement: Figure S18 — Concentrations of Cd over time. Concentration of Cd (ng/g of pellets) vs. time for each type of plastic (rows) at Coronado Cays (CC; left), and Shelter Island (SI; middle), and Nimitz Marine Facility (NMF; right). Note that vertical axes differ among graphs. Data were fit to the first-order approach to equilibrium model [26] using the exponential rise to maximum equation Ct = Ceq(1-e-kt), where Ct is the concentration at time t, Ceq is the predicted equilibrium concentration, and k is the rate constant. The horizontal dotted line denotes the predicted Ceq for each plastic type. Where no equation is given, the model could not be fit to the data and where no horizontal line is given the non-linear regression was not statistically significant (p>0.05). (TIFF) [file pone.0085433.s018.tiff]

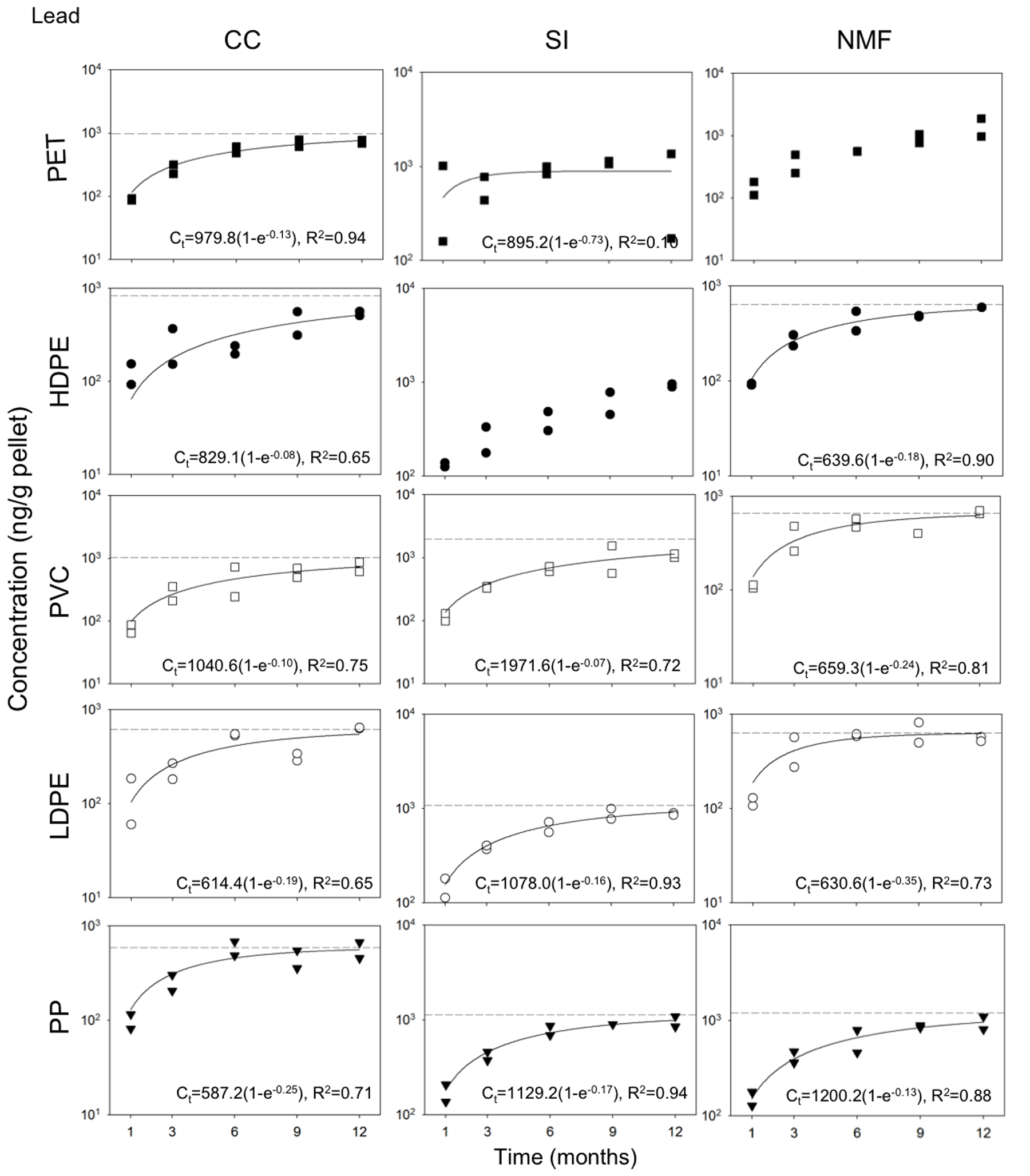

Supplement: Figure S19 — Concentrations of Pb over time. Concentration of Pb (ng/g of pellets) vs. time for each type of plastic (rows) at Coronado Cays (CC; left), and Shelter Island (SI; middle), and Nimitz Marine Facility (NMF; right). Note that vertical axes differ among graphs. Data were fit to the first-order approach to equilibrium model [26] using the exponential rise to maximum equation Ct = Ceq(1-e-kt), where Ct is the concentration at time t, Ceq is the predicted equilibrium concentration, and k is the rate constant. The horizontal dotted line denotes the predicted Ceq for each plastic type. Where no equation is given, the model could not be fit to the data and where no horizontal line is given the non-linear regression was not statistically significant (p>0.05). (TIFF) [file pone.0085433.s019.tiff]
