# Supplementary material for: Long-Term Sorption of Metals Is Similar among Plastic Types: Implications for Plastic Debris in Aquatic Environments
Source: PLoS One. 2014 Jan 15;9(1):e85433. doi: 10.1371/journal.pone.0085433 (PMC3893203; doi:10.1371/journal.pone.0085433)
Supplement: Table S1 — Concentrations of all terageted metals on individual samples for each time period among all plastic types at all three locations. Each individual table shows data for each of the five time periods. Concentrations of metals are given in ng/g pellet. (PDF) [file pone.0085433.s020.pdf]

Table S1. Concentrations of all targeted metals on individual samples for each time period among all plastic types at all three locations. Each individual table shows data for each of the five time periods. Concentrations of metals are given in ng/g pellet.

**1 month concentrations (ng/g pellet)**											
**Location**	**Polymer**	**Replicate**	**Al**	**Cr**	**Mn**	**Fe**	**Co**	**Ni**	**Zn**	**Cd**	**Pb**
CC	PET	1	4490	28	20072	14981	131	31	1181	nd	87
		2	57667	32	26920	19536	152	44	1570	nd	93
SI		1	74838	303	5315	89084	39	36	4007	1	1017
		2	270361	67	2423	34369	21	29	806	nd	159
NMF		1	22067	83	2894	47253	19	24	861	nd	182
		2	23551	54	2114	34314	15	19	432	nd	111
CC	HDPE	1	26923	35	38555	18752	184	26	1948	nd	153
		2	6260	16	34488	9503	173	nd	1737	nd	92
SI		1	13733	54	3468	21465	23	nd	758	nd	139
		2	9558	34	5437	11529	33	nd	642	nd	125
NMF		1	9620	87	2923	17671	12	nd	412	nd	90
		2	12591	34	2312	20620	11	nd	291	nd	94
CC	PVC	1	2895	15	24316	6809	143	26	1312	1	64
		2	4373	23	29035	12462	140	19	1148	nd	87
SI		1	10293	39	2032	16890	14	4	396	nd	100
		2	7537	44	2800	19024	19	8	456	nd	130
NMF		1	6243	30	1865	18275	10	8	228	1	105
		2	15332	59	1910	31943	12	10	374	nd	113
CC	LDPE	1	6649	13	28190	8388	125	21	1157	nd	60
		2	9772	28	42174	13406	221	48	2024	12	185
SI		1	49922	86	2217	40163	21	21	1076	1	180
		2	12242	49	2177	18997	15	9	440	nd	113
NMF		1	22794	53	2174	31124	12	17	491	1	107
		2	21084	67	2834	42025	16	19	533	nd	129
CC	PP	1	10201	31	23453	18146	138	29	1324	nd	116
		2	12961	19	30604	10510	155	27	1215	1	81
SI		1	27983	61	2222	27053	15	11	715	nd	137
		2	27017	98	3470	47504	29	20	1018	nd	208
NMF		1	20771	59	2640	39855	15	18	502	nd	128
		2	42020	123	2994	74414	25	37	886	nd	177

3 month concentrations (ng/g pellet)											
**Location**	**Polymer**	**Replicate**	**Al**	**Cr**	**Mn**	**Fe**	**Co**	**Ni**	**Zn**	**Cd**	**Pb**
CC	PET	1	9751	53	43958	27443	277	106	3310	nd	228
		2	52182	101	55448	41744	295	130	4442	nd	318
SI		1	22804	80	17820	37121	143	26	2134	nd	436
		2	99689	320	8962	81020	50	41	2720	nd	775
NMF		1	21635	83	6869	42240	45	24	1198	nd	252
		2	79488	241	5745	85370	31	43	2056	nd	495
CC	HDPE	1	51338	124	64906	71801	436	57	4776	nd	366
		2	22555	53	43725	30283	274	23	3139	nd	153
SI		1	42984	86	2741	40854	22	nd	797	nd	177
		2	11603	55	13852	25285	117	nd	1071	nd	334
NMF		1	12555	40	8890	28212	59	nd	680	nd	233
		2	86829	92	8200	61869	58	nd	1458	nd	305
CC	PVC	1	2512	14	40472	10028	262	97	2927	nd	209
		2	25394	43	65500	19728	291	142	4499	1	353
SI		1	35356	138	8837	28601	59	23	1030	nd	351
		2	10373	56	13506	22438	99	14	1120	nd	328
NMF		1	26678	87	7439	34162	34	14	984	1	259
		2	66121	228	6770	43964	20	16	1727	1	477
CC	LDPE	1	9929	76	47482	23468	308	165	3940	nd	268
		2	19657	26	53037	15886	325	145	3528	53	182
SI		1	26271	94	14120	37795	113	27	1469	1	369
		2	32961	117	13226	53153	116	31	1696	1	404
NMF		1	374773	359	8923	206321	86	119	2658	2	568
		2	32129	101	3023	63391	25	31	917	1	275
CC	PP	1	64970	73	43749	29718	265	74	2284	nd	205
		2	18101	60	64769	30030	333	154	4896	1	301
SI		1	40605	168	9523	37987	84	28	1424	1	375
		2	50818	164	9828	71966	91	37	1539	nd	460
NMF		1	90769	164	9299	103477	77	50	1336	1	361
		2	99462	236	7045	139200	63	71	1762	1	473

6 month concentrations (ng/g pellet)											
**Location**	**Polymer**	**Replicate**	**Al**	**Cr**	**Mn**	**Fe**	**Co**	**Ni**	**Zn**	**Cd**	**Pb**
CC	PET	1	12980	56	72903	32006	395	216	5929	2	482
		2	17363	88	43949	39284	267	144	4150	1	607
SI		1	24511	118	32220	49026	265	60	3483	nd	831
		2	67619	213	13171	101051	73	41	5717	nd	995
NMF		1	86024	214	7443	84547	51	50	3190	1	561
		2	36584	n/a	n/a	n/a	n/a	n/a	n/a	n/a	n/a
CC	HDPE	1	16423	52	23090	31881	173	nd	2330	nd	196
		2	9756	30	36818	24686	249	43	3051	nd	241
SI		1	11452	45	13523	22298	105	nd	1254	nd	305
		2	11182	43	24498	22868	245	nd	1935	nd	487
NMF		1	41243	167	15715	100529	156	nd	2157	nd	539
		2	20149	70	9349	45982	86	nd	1100	nd	335
CC	PVC	1	5799	35	40890	15088	225	102	3217	2	245
		2	6376	42	57394	17235	282	156	4707	2	723
SI		1	43106	113	19499	36530	116	26	2231	1	601
		2	31593	70	29705	27703	212	52	2444	nd	725
NMF		1	75788	230	9545	45626	33	19	2233	1	575
		2	33576	115	13801	39150	72	20	1800	2	466
CC	LDPE	1	7253	28	53692	17634	383	243	3969	22	528
		2	10239	48	61842	22997	455	239	6481	140	549
SI		1	35477	100	23327	47099	228	53	2582	3	560
		2	33004	125	32395	57225	311	74	3118	3	719
NMF		1	66746	243	9770	153571	99	87	2725	3	585
		2	86380	321	10616	185142	128	113	2619	3	617
CC	PP	1	92379	294	74984	62674	200	122	8363	3	683
		2	16614	49	74778	27573	450	251	6456	1	484
SI		1	34005	155	29505	70069	307	70	3350	1	689
		2	37994	132	40155	66474	395	91	4213	1	862
NMF		1	65109	232	6625	135716	68	75	2508	2	461
		2	79513	304	20441	178468	200	99	2411	1	792

9 month concentrations (ng/g pellet)											
**Location**	**Polymer**	**Replicate**	**Al**	**Cr**	**Mn**	**Fe**	**Co**	**Ni**	**Zn**	**Cd**	**Pb**
CC	PET	1	50098	144	70318	51495	355	258	6938	2	788
		2	30666	87	96713	38598	521	399	7951	2	614
SI		1	47114	209	34931	66593	245	68	4928	nd	1066
		2	58984	219	33667	65902	235	72	4813	nd	1136
NMF		1	80084	261	28032	81089	158	52	3163	2	769
		2	n/a	150	35596	63628	255	52	3514	1	1048
CC	HDPE	1	9170	34	41238	23403	380	99	3350	nd	313
		2	18733	79	80415	49282	658	394	6562	nd	558
SI		1	19897	71	17047	35208	180	nd	1837	nd	453
		2	63107	112	34241	57406	377	nd	3121	nd	779
NMF		1	21330	81	15782	51332	176	nd	1329	nd	486
		2	15712	68	17221	41774	183	nd	1513	nd	472
CC	PVC	1	7900	40	70361	16310	335	182	5252	2	494
		2	26388	94	94602	24671	325	256	7998	3	692
SI		1	40469	189	70099	52503	394	81	6473	1	1546
		2	9958	60	21843	22089	165	37	2090	1	564
NMF		1	12930	57	12943	26741	96	15	923	1	401
		2	n/a	n/a	n/a	n/a	n/a	n/a	n/a	n/a	n/a
CC	LDPE	1	6859	30	44621	19247	408	269	3960	3	285
		2	7446	36	66384	21795	545	390	5181	30	340
SI		1	46649	119	19375	72490	200	51	2367	nd	994
		2	38866	168	29858	80923	338	95	3398	4	770
NMF		1	118482	435	15900	259779	195	146	3232	3	815
		2	22595	95	15874	61780	161	48	1822	1	499
CC	PP	1	28346	92	74664	40144	410	341	6330	3	547
		2	38465	97	80318	38289	432	226	6765	2	355
SI		1	28200	139	33332	67563	390	79	3493	1	897
		2	26453	129	38350	66766	420	97	3720	1	899
NMF		1	97925	295	29610	182885	329	117	3067	2	889
		2	41746	149	32577	92713	343	82	3816	1	840

12 month concentrations (ng/g pellet)											
**Location**	**Polymer**	**Replicate**	**Al**	**Cr**	**Mn**	**Fe**	**Co**	**Ni**	**Zn**	**Cd**	**Pb**
CC	PET	1	51943	100	130201	40831	609	373	6100	1	775
		2	24528	107	96572	41577	508	361	5504	3	690
SI		1	16727	79	2414	34936	21	19	789	nd	171
		2	37581	184	53682	55945	347	87	4698	nd	1360
NMF		1	107027	394	28305	111896	120	61	3708	1	965
		2	101483	454	77459	90856	315	86	8384	6	1873
CC	HDPE	1	33708	119	92279	80874	829	424	6741	nd	563
		2	34774	150	45143	100823	339	85	6310	nd	506
SI		1	24310	105	44751	53444	473	13	3779	nd	888
		2	17052	87	44727	47567	510	28	4019	nd	962
NMF		1	n/a	n/a	n/a	n/a	n/a	nd	n/a	nd	594
		2	17220	79	21173	49516	288	nd	2573	nd	n/a
CC	PVC	1	36912	146	113378	29023	332	168	6560	3	609
		2	20365	97	102389	26721	307	199	4613	2	866
SI		1	32898	138	40922	32040	218	54	3599	1	1009
		2	60428	157	21639	39395	122	32	2468	nd	1151
NMF		1	31273	124	17156	38052	86	19	1322	2	649
		2	20093	95	33301	29867	188	30	2873	1	702
CC	LDPE	1	24116	87	75167	67117	625	442	6099	8	629
		2	35516	96	96344	63077	838	581	7902	30	644
SI		1	40154	175	18359	97968	172	62	3718	2	893
		2	54307	258	7000	157921	84	60	3697	2	849
NMF		1	36895	149	19468	91565	211	67	2012	3	573
		2	15654	79	14287	47382	203	45	1615	1	515
CC	PP	1	29161	112	75361	44371	408	260	5175	2	668
		2	21679	69	53731	37579	279	148	2283	1	453
SI		1	58400	321	14513	152981	180	86	4597	2	1092
		2	40704	159	21242	91806	249	73	2411	1	848
NMF		1	105266	394	16741	249439	192	133	3536	4	1099
		2	32013	131	20091	78776	264	75	3701	4	806
