# Supplementary material for: Long-Term Sorption of Metals Is Similar among Plastic Types: Implications for Plastic Debris in Aquatic Environments
Source: PLoS One. 2014 Jan 15;9(1):e85433. doi: 10.1371/journal.pone.0085433 (PMC3893203; doi:10.1371/journal.pone.0085433)
Supplement: Table S2 — Two-way Analysis of Variance (ANOVA) tables for each metal individually (in order of molecular weight) between polymers and locations within San Diego Bay for each of the five time periods (1, 3, 6, 9 and 12 months). SNK results are given in order from highest to lowest concentration for each contaminant group among polymers and locations. (PDF) [file pone.0085433.s021.pdf]

Table S2. Two-way Analysis of Variance (ANOVA) Tables for Each Metal Individually (in order of molecular weight) Between Polymers and Locations Within San Diego Bay for Each of Five Time Periods (1, 3, 6, 9, and 12 months). SNK results are given in order from highest to lowest concentration for each contaminant group among polymers and locations.

**Al(Aluminum) 2-way ANOVA**		**1 month**			**3 month**			**6 month**			**9 month**			**12 month**		
**Source**	**df**	**MS**	**F**	**P**	**MS**	**F**	**P**	**MS**	**F**	**P**	**MS**	**F**	**P**	**MS**	**F**	**P**
Polymer	4	1.648	1.8	0.2	0.864	1.1	0.4	0.958	2.1	0.2	1.525	2.9	0.1	0.433	0.8	0.5
Location	2	3.931	15.1	0.003	2.854	3.7	0.04	4.986	22.7	<0.001	1.552	5.0	0.02	0.097	0.6	0.6
Poly × Loc	8	0.894	3.4	0.02	0.421	pooled		0.450	2.1	0.1	0.526	1.7	0.2	0.519	3.2	0.03
Residual	15	0.260			0.958			0.220			0.309			0.163		
**Cochran's Test**		C=0.2725 ns			C=0.2100 ns			C=0.4470 ns			C=0.2966 ns			C=0.2905 ns		
**SNK:**																
**Polymer**		N/A			ns			ns			ns			N/A		
**Location**		N/A			CC=SI=NMF			CC<SI<NMF			CC<SI=NMF			N/A		
**Poly(Loc)**		CC(PVC=PET=LDPE=PP=HDPE)<br>SI(PVC=HDPE=LDPE=PP<PET)<br>NMF(PVC=HDPE=LDPE=PET=PP)			N/A			ns			ns			CC(PP=PVC=LDPE=HDPE=PET)<br>SI(HDPE=PET=PVC=LDPE=PP)<br>NMF(HDPE=LDPE=PVC=PP=PET)		
**Cr(Chromium) 2-way ANOVA**		**1 month**			**3 month**			**6 month**			**9 month**			**12 month**		
**Source**	**df**	**MS**	**F**	**P**	**MS**	**F**	**P**	**MS**	**F**	**P**	**MS**	**F**	**P**	**MS**	**F**	**P**
Polymer	4	0.488	2.6	0.06	0.533	1.5	0.2	1.044	5.5	0.003	0.906	3.7	0.02	0.306	0.6	0.6
Location	2	3.459	18.7	<0.001	2.398	6.7	0.01	4.081	21.6	<0.001	1.642	6.8	0.005	0.465	3.9	0.04
Poly × Loc	8	0.114	pooled		0.344	pooled		0.166	pooled		0.293	pooled		0.475	4.0	0.01
Residual	15	0.223			0.367			0.201			0.215			0.120		
**Cochran's Test**		C=0.3387 ns			C=0.1742 ns			C=0.5246*			C=0.3595 ns			C=0.3383 ns		
**SNK:**																
**Polymer**		ns			ns			HDPE=PVC=LDPE=PET=PP			HDPE=PVC=LDPE=PP=PET			N/A		
**Location**		CC<NMF=SI			CC<SI=NMF			CC<SI<NMF			CC<NMF=SI			N/A		
**Poly(Loc)**		N/A			N/A			N/A			N/A			CC(PP=LDPE=PET=PVC=HDPE)<br>SI(HDPE=PET=PVC=LDPE=PP)<br>NMF(HDPE=LDPE=PVC=PP=PET)		
**Mn(Manganese) 2-way ANOVA**		**1 month**			**3 month**			**6 month**			**9 month**			**12 month**		
**Source**	**df**	**MS**	**F**	**P**	**MS**	**F**	**P**	**MS**	**F**	**P**	**MS**	**F**	**P**	**MS**	**F**	**P**
Polymer	4	0.130	2.1	0.1	0.017	0.1	0.98	0.234	2.0	0.1	0.323	3.3	0.03	0.3398	0.8	0.6
Location	2	19.04	300	<0.001	11.30	71.4	<0.001	6.316	52.6	<0.001	3.966	40.0	<0.001	6.353	14.1	<0.001
Poly × Loc	8	0.075	pooled		0.131	pooled		0.107	pooled		0.102	pooled		0.435	pooled	
Residual	15	0.058			0.173			0.128			0.098			0.449		
**Cochran's Test**		C=0.3580 ns			C=0.5061*			C=0.3319 ns			C=0.4646 ns			C=0.7142**		
**SNK:**																
**Polymer**		ns			ns			ns			LDPE=HDPE=PVC=PP=PET			ns		
**Location**		NMF=SI<CC			NMF<SI<CC			NMF<SI<CC			NMF<SI<CC			SI=NMF<CC		
**Poly(Loc)**		N/A			N/A			N/A			N/A			N/A		

Fe(Iron) 2-way ANOVA		1 month			3 month			6 month			9 month			12 month		
**Source**	**df**	**MS**	**F**	**P**	**MS**	**F**	**P**	**MS**	**F**	**P**	**MS**	**F**	**P**	**MS**	**F**	**P**
Polymer	4	0.733	5.1	0.004	0.659	2.2	0.2	1.016	4.4	0.04	0.915	3.6	0.06	0.941	2.4	0.1
Location	2	2.593	18.1	<0.001	2.166	11.6	<0.001	3.830	50.9	<0.001	1.816	12.2	<0.001	0.344	3.8	0.05
Poly × Loc	8	0.130	pooled		0.295	1.6	0.2	0.229	3.1	0.03	0.252	1.7	0.2	0.397	4.4	0.007
Residual	15	0.151			0.187			0.075			0.148			0.091		
**Cochran's Test**		C=0.2005 ns			C=0.2482 ns			C=0.2989 ns			C=0.4632 ns			C=0.4887*		
**SNK:**																
**Polymer**		PVC=HDPE=LDPE=PP=PET			ns			N/A			ns			N/A		
**Location**		CC<SI=NMF			CC<SI<NMF			N/A			CC<SI=NMF			N/A		
**Poly(Loc)**		N/A			ns			CC(HDPE=PVC=LDPE=PP=PET)<br>SI(HDPE=PVC=LDPE=PP=PET)<br>NMF(PVC=HDPE=PET=PP=LDPE)						CC(PVC=PP=PET=LDPE=HDPE)<br>SI(PVC=PET=HDPE<PP=LDPE)<br>NMF(PVC=HDPE=LDPE=PET=PP)		
**Co(Cobalt) 2-way ANOVA**		**1 month**			**3 month**			**6 month**			**9 month**			**12 month**		
**Source**	**df**	**MS**	**F**	**P**	**MS**	**F**	**P**	**MS**	**F**	**P**	**MS**	**F**	**P**	**MS**	**F**	**P**
Polymer	4	0.108	1.5	0.2	0.155	0.8	0.5	0.534	2.8	0.1	0.320	3.6	0.02	0.581	1.5	0.2
Location	2	16.24	223	<0.001	9.553	49.5	<0.001	4.209	22.0	<0.001	1.785	19.9	<0.001	2.818	7.4	0.003
Poly × Loc	8	0.085	pooled		0.160	pooled		0.169	pooled		0.109	pooled		0.364	pooled	
Residual	15	0.066			0.211			0.203			0.080			0.387		
**Cochran's Test**		C=0.2152 ns			C=0.4315 ns			C=0.2746 ns			C=0.3159 ns			C=0.6834**		
**SNK:**																
**Polymer**		ns			ns			ns			PVC=PET=LDPE=HDPE=PP			ns		
**Location**		NMF<SI<CC			NMF<SI<CC			NMF<SI=CC			NMF<SI<CC			SI=NMF<CC		
**Poly(Loc)**		N/A			N/A			N/A			N/A			N/A		
**Ni(Nickel) 2-way ANOVA**		**1 month**			**3 month**			**6 month**			**9 month**			**12 month**		
**Source**	**df**	**MS**	**F**	**P**	**MS**	**F**	**P**	**MS**	**F**	**P**	**MS**	**F**	**P**	**MS**	**F**	**P**
Polymer	4	23.44	23.4	<0.001	22.06	7.6	0.01	39.51	37.8	<0.001	23.64	4.6	0.03	8.579	1.9	0.2
Location	2	3.775	3.78	0.04	17.25	117	<0.001	7.916	7.6	0.003	25.61	157	<0.001	20.40	88	<0.001
Poly × Loc	8	0.754	pooled		2.920	19.9	<0.001	0.596	pooled		5.115	31.3	<0.001	4.484	19.3	<0.001
Residual	15	1.132			0.147			1.285			0.163			0.2319		
**Cochran's Test**		C=0.9087**			C=0.4180 ns			C=0.9539**			C=0.3873 ns			C=0.3712 ns		
**SNK:**																
**Polymer**		HDPE<PVC=LDPE=PP=PET			N/A			HDPE<PVC=PET=PP=LDPE			N/A			N/A		
**Location**		SI=NMF<CC			N/A			NMF=SI<CC			N/A			N/A		
**Poly(Loc)**		N/A			CC(HDPE<PP=PVC=PET=LDPE)<br>SI(HDPE<PVC=LDPE=PP=PET)<br>NMF(HDPE<PVC=PET=PP=LDPE)			N/A			CC(HDPE=PVC=PP=PET=LDPE)<br>SI(HDPE<PVC=LDPE=PET=PP)<br>NMF(HDPE<PVC<PET=LDPE=PP)			CC(PVC=HDPE=PP=PET=LDPE)<br>SI(HDPE=PET=PVC=LDPE=PP)<br>NMF(HDPE<PVC=LDPE=PET=PP)		
**Zn(Zinc) 2-way ANOVA**		**1 month**			**3 month**			**6 month**			**9 month**			**12 month**		
**Source**	**df**	**MS**	**F**	**P**	**MS**	**F(p)**	**P**	**MS**	**F(p)**	**P**	**MS**	**F**	**P**	**MS**	**F**	**P**
Polymer	4	0.459	2.5	0.07	0.195	1.8	0.2	0.682	12.9	<0.001	0.496	2.5	0.1	0.059	0.2	0.9
Location	2	3.195	17.5	<0.001	3.146	29.3	<0.001	1.216	23	<0.001	2.724	29	<0.001	1.295	6.5	0.009
Poly × Loc	8	0.202	pooled		0.083	pooled		0.047	pooled		0.199	2.1	0.1	0.344	1.7	0.2
Residual	15	0.172			0.121			0.056			0.094			0.198		
**Cochran's Test**		C=0.4996*			C=0.3127 ns			C=0.2691 ns			C=0.4530 ns			C=0.5359*		
**SNK:**																
**Polymer**		ns			ns			HDPE<PVC=LDPE=PP=PET			ns			ns		
**Location**		NMF<SI<CC			NMF=SI<CC			NMF=SI<CC			NMF<SI<CC			NMF=SI<CC		
**Poly(Loc)**		N/A			N/A			N/A			ns			ns		

Cd(Cadmium)<br>2-way ANOVA		1 month			3 month			6 month			9 month			12 month		
**Source**	**df**	**MS**	**F**	**P**	**MS**	**F**	**P**	**MS**	**F**	**P**	**MS**	**F**	**P**	**MS**	**F**	**P**
Polymer	4	3.247	3.2	0.03	8.308	9.3	<0.001	15.10	13.4	0.001	9.865	9.2	0.004	10.97	8.5	0.006
Location	2	0.759	0.74	0.5	1.673	1.9	0.2	6.029	38.4	<0.001	5.052	11.2	0.001	3.457	9.4	0.002
Poly × Loc	8	0.671	pooled		0.712	pooled		1.128	7.2	<0.001	1.078	2.39	0.07	1.297	3.5	0.02
Residual	15	1.224			0.985			0.157			0.452			0.370		
**Cochran's Test**		C=0.6262**			C=0.7126**			C=0.7231**			C=0.3854 ns			C=0.3609 ns		
**SNK:**																
**Polymer**		HDPE=PET=PP=PVC=LDPE			HDPE=PET=PP=PVC=LDPE			N/A			HDPE<PET=PP=PVC=LDPE			N/A		
**Location**		ns			ns			N/A			SI<NMF=CC			N/A		
**Poly(Loc)**		N/A			N/A			CC(HDPE<PET=PP=PVC<LDPE<br>SI(HDPE=PET<PVC=PP<LDPE)<br>NMF(HDPE<PET=PP=PVC=LDPE)			ns			CC(HDPE<PP=PET=PVC<LDPE)<br>SI(HDPE=PET<PVC=PP=LDPE)<br>NMF(HDPE<PVC=LDPE=PET=PP)		
**Pb(Lead)<br>2-way ANOVA**		**1 month**			**3 month**			**6 month**			**9 month**			**12 month**		
**Source**	**df**	**MS**	**F**	**P**	**MS**	**F**	**P**	**MS**	**F**	**P**	**MS**	**F**	**P**	**MS**	**F**	**P**
Polymer	4	0.272	1.4	0.3	0.153	1.5	0.2	0.480	7.0	<0.001	0.303	2.5	0.1	0.037	0.2	0.9
Location	2	0.839	4.3	0.03	0.501	4.9	0.02	0.402	5.9	0.009	0.915	11.2	0.001	0.238	1.2	0.3
Poly × Loc	8	0.183	pooled		0.060	pooled		0.049	pooled		0.123	1.5	0.2	0.223	pooled	
Residual	15	0.202			0.125			0.079			0.082			0.183		
**Cochran's Test**		C=0.5674*			C=0.2050 ns			C=0.4984*			C=0.4155 ns			C=0.7853**		
**SNK:**																
**Polymer**		ns			ns			HDPE<PVC=LDPE=PP=PET			ns			ns		
**Location**		CC=NMF=SI			CC<NMF=SI			CC=NMF=SI			CC<NMF<SI			ns		
**Poly(Loc)**		N/A			N/A			N/A			ns			ns		
